# Advancements in In-Situ Monitoring Technologies for Detecting Process-Induced Defects in the Directed Energy Deposition Process: A Comprehensive Review

**DOI:** 10.3390/ma18184304

**Published:** 2025-09-14

**Authors:** Md Jonaet Ansari, Anthony Roccisano, Elias J. G. Arcondoulis, Christiane Schulz, Thomas Schläfer, Colin Hall

**Affiliations:** 1 Future Industries Institute, University of South Australia, Mawson Lakes, SA 5095, Australia; 2 The Australian Research Council (ARC), Industrial Transformation Training Centre in Surface Engineering for Advanced Materials (SEAM), Hawthorn, VIC 3122, Australia; 3 School of Civil Aerospace and Design Engineering, University of Bristol, Bristol BS8 1TH, UK; 4 LaserBond Ltd., Cavan, SA 5094, Australia

**Keywords:** directed energy deposition, defects, in-situ monitoring technique

## Abstract

Laser-based directed energy deposition for metallic materials (DED-LB/M) is a versatile additive manufacturing (AM) technique that facilitates the deposition of advanced protective coatings, the refurbishment of degraded components, and the fabrication of intricate metallic structures. Despite the technological advancements and potential, the presence of process-induced defects poses significant challenges to the repeatability and stability of the DED-LB/M process, limiting its widespread application, particularly in industries requiring high-quality products. In-situ process monitoring stands out as a key technological intervention, offering the possibility of real-time defect detection to mitigate these challenges. Focusing on the DED-LB/M process, this review provides a comparative analysis of various in-situ monitoring techniques and their effectiveness in identifying process-induced defects. The review categorises different sensing methods based on their sensor data format, utilised data processing techniques, and their ability to detect both surface and internal defects within the fabricated structures. Furthermore, it compares the capabilities of these techniques and offers a critical analysis of their limitations in defect detection. This review concludes by discussing the major challenges that remain in implementing in-situ defect detection in industrial practice and outlines key future directions necessary to overcome them.

## 1. Introduction

Additive manufacturing (AM) has revolutionised the production of coating and three-dimensional (3D) metallic structures through its layer-by-layer deposition process [1]. This approach offers several key advantages over traditional subtractive manufacturing methods, including enhanced design flexibility for complex geometries [2], reduced material waste [3], shorter lead times for prototyping and small-batch production [4], and the ability to create lightweight structures with optimised material distribution [5]. These advantages have led to the widespread adoption of AM across various industries, including automotive [6], aerospace [7,8], medical [9], and manufacturing [10].

Laser cladding (LC) is a buildup welding process that uses a high-power laser to create a metallurgical bond between a coating material and a substrate [11]. This process involves feeding a coating material, typically in powder or wire form, into a melt pool generated by a focused laser beam on the substrate surface [12,13]. The precise control of laser energy allows for minimal dilution between the coatings and substrate materials, resulting in high-quality, metallurgically bonded coatings with excellent wear and corrosion resistance properties [14]. 

Directed energy deposition (DED) extends the capabilities of laser cladding by enabling the fabrication of entire 3D structures, not just surface coatings [15]. This process has acquired considerable attention within the manufacturing sector due to its capacity to produce large metallic components at high speeds [16]. The versatility of DED, evidenced by its applicability in both new part production and the refurbishment of existing structures, highlights its increasing importance in modern manufacturing practices [17].

To standardise terminology, the latest ISO/ASTM Standard (ISO/ASTM 52900:2021) recommends the use of DED-LB/M, where ‘LB’ denotes laser beam and ‘M’ signifies metallic materials [18]; the term DED-LB/M is primarily employed to describe this process. The inherent complexity of the DED-LB/M process, characterised by rapid thermal cycles and dynamic melt pool behaviour, poses significant challenges in maintaining consistent part quality throughout the fabrication process. These challenges arise from the complex interactions between various process parameters, such as laser power, powder feed rate, scanning speed, and material-specific factors like potential powder impurities. It is important to note that defects can still form even with optimised process parameters due to the dynamic thermal behaviour inherent to the DED-LB/M process [19]. Such defects can adversely affect the mechanical properties and overall quality of the manufactured structures [20]. Traditional monitoring techniques primarily target surface-level defects but often overlook subsurface anomalies that can compromise part integrity [21]. This limitation underscores the urgent requirement for advanced monitoring approaches to detect surface and subsurface defects in real-time during the DED-LB/M process.

The development of effective in-situ defect detection methods has become increasingly crucial for several reasons. Early detection of imperfections and flaws in manufactured components is essential for maintaining product quality, as timely identification allows for swift corrective actions, preventing defects from propagating through subsequent stages of production. By identifying defects during the fabrication rather than post-production, manufacturers can significantly reduce inspection costs and time while enhancing overall part quality. Furthermore, real-time monitoring systems provide valuable insights into process dynamics, enabling timely adjustments to mitigate defects and optimise manufacturing processes. Crack source localisation has emerged as a critical aspect of quality control in DED-LB/M processes, as accurate localisation of cracks provides invaluable information about the spatial distribution of defects within fabricated structures [22]. This enables more targeted corrective actions and improves overall structural integrity. Understanding where cracks initiate and propagate can also inform adjustments to process parameters to minimise defect formation. 

Driven by the increasing need for enhanced quality assurance in AM processes, extensive research efforts have been directed toward developing in-situ monitoring strategies for detecting process-induced defects in the DED-LB/M process. This review presents a comprehensive analysis of recent advancements in this domain, structured around three key themes:

1. The nature and formation of process-induced defects,
2. Current advancements in in-situ monitoring approaches for defect detection, and
3. Advanced methods for precise localisation of defects within DED-LB/M fabricated structures.

To systematically address these topics, the review is organised into four main sections. It delves into the fundamental principles underlying DED-LB/M, the critical process parameters influencing part quality, and the range of materials commonly used in DED-LB/M manufacturing. This section concludes with a detailed discussion on process-induced defects in DED-LB/M, exploring their types, causes, and the consequential effects on the mechanical properties and overall performance of structures manufactured via DED-LB/M.

Section 3.2 explores the wide array of in-situ process monitoring methods currently employed in AM processes, emphasising their application in identifying process-induced defects during the DED-LB/M process. This section is further divided into subsections covering optical sensing, thermal sensing, spectra sensing, and acoustic emission. By examining various technologies, this section evaluates their effectiveness, limitations, and potential for integration into DED-LB/M systems.

Section 3.3 reviews existing knowledge gaps related to monitoring and identifying defects during the DED-LB/M process. By highlighting areas where further investigation is needed, this section aims to stimulate future research directions and inspire innovative solutions to the challenges faced in DED-LB/M manufacturing.

## 2. Directed Energy Deposition Background

Among the various AM technologies for metal structures, laser-based powder bed fusion for metallic materials (PBF-LB/M) and DED-LB/M stand out as the most widely used methods [23]. The PBF-LB/M process fabricates complex 3D structures by selectively melting and fusing metal powder particles in accordance with a digital model [24]. During this process, a thin layer of metal powder is uniformly distributed over a build platform. A focused energy source, such as a laser, is then used to selectively melt the powder at designated locations as specified by the model. This process of layering and selective melting continues in a repetitive manner until the entire structure is formed [25]. PBF-LB/M’s versatility enables the creation of intricate designs, allows for significant weight reduction in structures, and offers compatibility with a wide range of metal materials [26]. Due to the limited melt pool size, the average layer thickness is typically between 20 and 100 µm, offering excellent dimensional accuracy for structures [27]. Build size limitations and production efficiency remain significant hurdles for widespread PBF adoption, particularly for larger components or high-volume production scenarios.

In contrast, DED-LB/M utilises a high-powered laser beam to melt metal powder onto a metallic substrate. The laser generates a melt pool where the injected powder feedstock fuses with the substrate, forming a metallurgical bond [28]. By synchronising the movements of both the laser optics and the powder feeding nozzle, this method can produce uniform coatings and complex 3D structures [14,29]. Compared to PBF-LB/M, DED-LB/M offers higher manufacturing efficiency, a more comprehensive process parameter window, and excellent structural density and metallurgical bonding in fabricated structures [30]. For this reason, the report focuses solely on DED-LB/M structural fabrication, and the subsections below describe the history, current use, and remaining challenges of the technique in such applications.

## 3. Directed Energy Deposition Process

### 3.1. Principle of DED-LB/M Process

**Figure 1:**
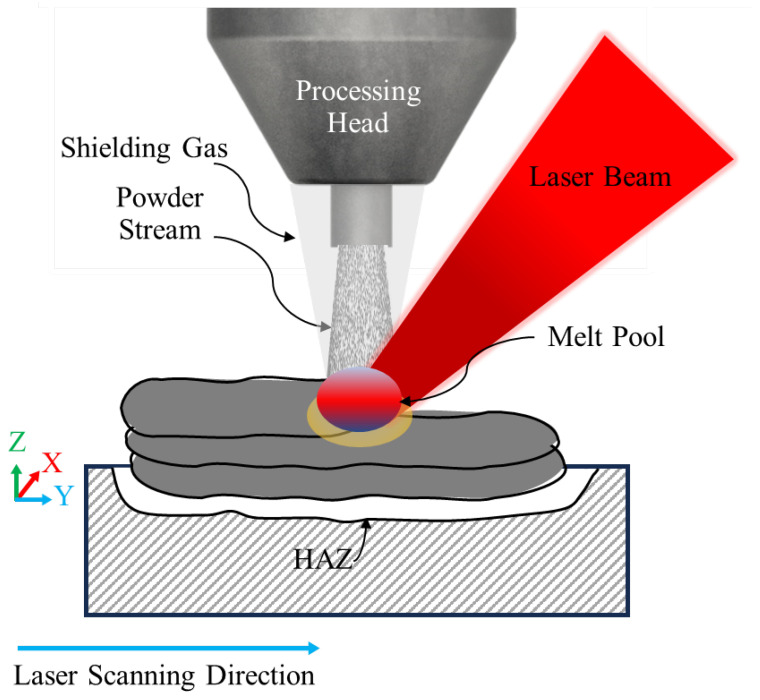
Schematic representation of the DED-LB/M process, illustrating the use of metallic powder as a feedstock material, adapted from [36].

The DED-LB/M process employs a focused laser beam to generate a localised melt pool on the workpiece surface. Concurrently, metal powder is introduced into this melt pool via a carrier gas through a specialised feeding nozzle [31]. The heat generated by the laser melts the metallic powder, which then rapidly solidifies, establishing a strong metallurgical bond with the substrate [32]. The deposition process is controlled by manipulating either the processing head (containing laser optics and the powder feeding nozzle) or the substrate, or both. Typically, a robot arm governs these movements to ensure uniform coatings or the precise construction of 3D structures [33]. Common laser types used in DED-LB/M include Nd:YAG (neodymium-doped yttrium aluminum garnet), CO_2_, diode, and fibre lasers [34]. The injected powder melted by the laser creates a small, localised molten pool on the substrate surface. As this molten material rapidly cools and solidifies, it forms a thin layer that is metallurgically bonded to the underlying substrate or previously deposited layers. This process of melting, deposition, and solidification occurs continuously as the laser and powder feed nozzle move, allowing for the layer-by-layer fabrication of complex 3D structures or the addition of material to existing structures [35]. Figure 1 presents a schematic diagram of the DED-LB/M process principle using metallic powder as a feedstock material.

**Figure 2:**
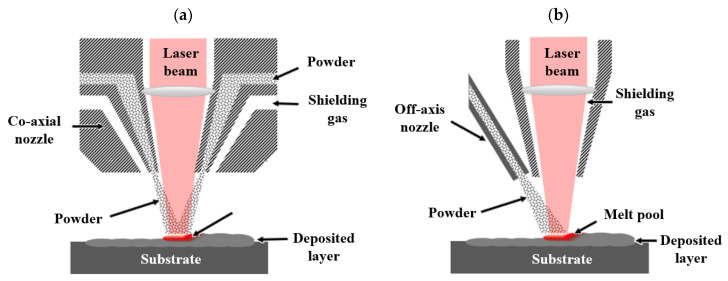
Schematic representation of powder feeding methodologies in the DED-LB/M process, adapted from [17]. (**a**) Co-axial powder injection, where the powder is fed symmetrically around the laser beam. (**b**) Off-axis powder injection, where the powder is delivered from a single direction relative to the laser beam.

As illustrated in Figure 2, there are primarily two types of powder feeding nozzles currently employed in DED-LB/M systems: off-axis nozzles and co-axial nozzles [17]. Off-axis nozzles inject powder into the melt pool from a single direction using one nozzle, whereas co-axial nozzles feed the powder symmetrically around the heat source. Off-axis nozzles are particularly advantageous in situations where certain alloys require specific powder injection angles relative to the laser beam to achieve optimal final quality [37]. In contrast, co-axial nozzles offer the benefit of directional independence, thereby providing greater flexibility in the build-up procedure. Furthermore, co-axial nozzles generally demonstrate higher efficiency compared to off-axis nozzles, enabling improved powder utilisation [37,38].

To control the powder feed rate and facilitate its delivery into the melt pool, DED-LB/M systems employ a dedicated powder feeder mechanism. The powder is transported to the melt pool via a carrier gas, while a separate shielding gas protects the processing zone from oxidation [39]. Argon is frequently selected for both carrier and shielding purposes due to its relative cost-effectiveness compared to alternative gases such as helium. Additionally, argon’s higher density compared to air ensures it remains in the process zone for an extended period, providing more effective shielding [39].

#### 3.1.1. Process Parameters

**Figure 3:**
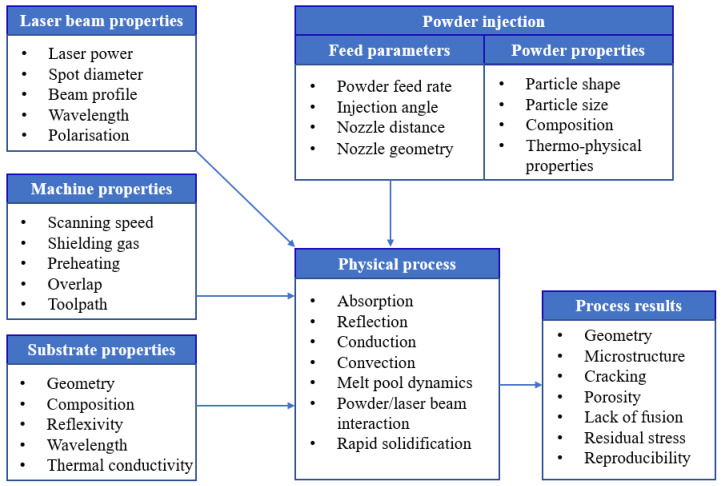
Different process parameters and variables in DED-LB/M process.

The DED-LB/M process involves a complex interplay of numerous process variables that significantly influence the quality of manufactured structures. Figure 3 illustrates the various process parameters and variables inherent to the DED-LB/M process. Among these, laser power, scanning speed, and energy density are identified as the most critical parameters affecting the quality and properties of the fabricated structures [40]. The precise control and optimisation of these parameters are important for attaining the specified structural geometry and minimising defects in fabricated structures [13].

Laser power is defined as the amount of energy delivered by the laser system during the DED-LB/M process and is recognised as a critical parameter affecting the quality and characteristics of the deposited material [41,42,43]. The selection of appropriate laser power is essential, as it directly impacts the thermal dynamics of the deposition process. Insufficient laser power can lead to incomplete melting or inadequate adhesion between layers, resulting in defects such as porosity [44,45]. Conversely, excessive laser power may cause overheating, which can result in undesirable thermal stresses and potential cracking within the deposited layers [46,47]. Therefore, optimising laser power is vital for achieving desired material properties while minimising defects during fabrication. Research-scale DED-LB/M systems typically operate with laser powers ranging from 200 W to 2 kW, whereas commercial systems commonly utilise powers between 2 kW and 20 kW. For instance, the Fraunhofer Institute for Laser Technology (ILT) has developed a research unit capable of operating at up to 20 kW, demonstrating the current upper limits of high-power DED-LB/M applications in industrial settings [48]. This capability highlights the potential for enhanced performance and efficiency in specialised applications that demand higher energy input.

Scanning speed denotes to the velocity at which the laser beam moves across the surface of the substrate [49]. It determines the laser-material interaction time for a particular spot area and therefore can be a dominant factor for the final characteristics of depositing metallic alloys [50]. Lower scanning speeds lead to a larger melt pool and slower cooling rates, potentially improving layer fusion and deposit density. However, excessively low speeds can cause overheating, leading to residual stress, and part distortion [51,52]. Higher scanning speeds produce smaller melt pool and faster cooling rates, potentially yielding finer microstructures and improved mechanical properties. However, if too high, insufficient melting can occur, leading to lack of fusion (LoF) defects. The optimal scanning speed often depends on other process parameters and must be carefully balanced to achieve desired part qualities [53].

Laser beam spot size denotes the diameter of the laser beam at its focal point, usually ranging from a few hundred micrometres to 1–6 mm in DED-LB/M processes [54]. This parameter significantly influences the energy distribution and concentration on the substrate surface, affecting melting efficiency and adhesion quality of deposited material. The power profile, which describes energy distribution across the spot size, is equally crucial. Common profiles include uniform top-hat distributions, ensuring even energy delivery, and Gaussian profiles, concentrating energy at the centre [55,56]. The combination of spot size and power profile directly impacts the power density, which determines how the laser interacts with the material. 

Energy density, expressed as $E\;=\;\frac{P}{d\;\cdot \;v}(W/{\mathrm{mm}}^{2}),\;$where *P* denotes the laser power (W), *d* represents the laser spot diameter (mm), and *v* signifies the scanning speed (mm/s), is a key parameter in DED-LB/M. It governs the energy required to melt the powder feedstock and substrate, significantly influencing melt pool temperature and size throughout the process [44]. Excessive or insufficient energy density can generate various process-induced defects in the manufactured structures. Therefore, it is necessary to select optimal process parameter settings (e.g., laser power, scanning speed, energy density) with respect to other process variables to avoid defect formation and ensure high-quality DED-LB/M manufactured components.

Powder feed rate refers to the rate at which metal powder is delivered into the melt pool created by the laser beam [57]. The powder feed rate is typically measured in grams per minute (g/min) and is controlled by specialised powder feeding systems [58]. In DED-LB/M applications, the powder feed rate directly affects the deposition rate, layer thickness, and overall quality of the fabricated structures. A higher powder feed rate can increase deposition rates and produce thicker layers, potentially improving productivity. However, excessively high feed rates leads to incomplete melting of powder particles, leading to LoF defects in the deposited material [20]. Conversely, a lower powder feed rate allows for more complete melting of the powder particles, potentially improving the density and fusion of the deposited material [59]. However, if the feed rate is too low, it can result in insufficient material deposition, affecting the geometry and properties of the fabricated part. A careful balance between powder feed rate and incident energy density is therefore required to achieve an adequate DED-LB/M process for a specific material system and application.

Even when process parameters are optimised, the DED-LB/M process remains susceptible to defects due to dynamic process conditions. These dynamic conditions, such as fluctuations in powder feed rate, variations in substrate temperature, or changes in local heat dissipation, can lead to inconsistencies in the deposition process. Furthermore, constant heat input using optimal process parameter settings can cause overheating and lack of adherence to the base material, potentially leading to the fabrication of defective structures and reducing overall part quality. This highlights the complexity of the DED-LB/M process and the ongoing challenges in achieving consistent, high-quality structures, even with optimised parameters.

#### 3.1.2. DED-LB/M Materials

The selection and characteristics of materials are fundamental to the success of the DED-LB/M process, as they directly impact the integrity, performance, and applicability of the fabricated structures. Indeed, DED-LB/M offers the flexibility to process a broad spectrum of materials, including metals and composites, establishing it as a highly adaptable method for various industrial applications [60,61,62]. Among these, metallic alloys are particularly well-suited for DED-LB/M due to their ease of process control and satisfactory final properties. Common materials used in DED-LB/M include Fe-based alloys such as stainless steels and tool steels, Co-based alloys like Stellite™, Ni-based self-fluxing (SF) alloys, Ti-based alloys, and Al-based alloys [41,63]. Each material category offers specific advantages, tailored to different applications and operating conditions.

For instance, Co-based alloys are frequently used in DED-LB/M due to their exceptional wear and corrosion resistance at elevated temperatures [64]. On the other hand, Ni-based alloys have gained significant attention for their superior wear and corrosion resistance and reasonable cost [65]. Fe-based alloys offer a cost-effective solution with good wear and corrosion resistance, high machinability, and excellent mechanical properties including high toughness and hardness [60,66]. Meanwhile, Ti-based alloys are crucial for medical and aerospace applications, whereas Al-based alloys are suitable for lightweight, high-strength components [67,68].

Ni-based MMCs, are engineered materials that combine a nickel alloy matrix with reinforcing phases to enhance mechanical, thermal, and corrosion-resistant properties [69]. These composites are particularly well-suited for high-temperature applications due to nickel’s inherent heat resistance and oxidation stability [70]. The choice of material composition in MMCs is primarily driven by specific application requirements, such as mechanical performance, corrosion resistance, and operating temperature. Common reinforcements used in these composites include carbides, nitrides, oxides, and intermetallics, which significantly improve the strength, stiffness, and wear resistance of the nickel matrix [71]. This combination of properties has resulted in their widespread utilisation in mining, automotive, and energy production industries, where materials must withstand extreme operating conditions while maintaining excellent high-temperature strength, corrosion resistance, and wear resistance [72].

#### 3.1.3. Process-Induced Defects 

Despite the numerous advantages of the DED-LB/M process, including its ability to create large and complex components, the presence of process-induced defects including porosity and cracking significantly undermines its reliability and trustworthiness [73]. Traditional non-destructive testing (NDT) methods, including dye penetrant inspection, ultrasonic testing, and radiographic testing, are commonly employed in industry to detect defects in large structures [74]. However, these methods are typically conducted after fabrication, which limit their effectiveness in identifying defects in real-time. Furthermore, while some techniques can detect subsurface flaws, others are restricted to surface defect detection, potentially overlooking critical internal defects [75,76]. The X-ray computed tomography (CT) technique is effective for detailed internal defect analysis but faces significant limitations when applied to large metallic parts, rendering it impractical for many industrial-scale DED-LB/M structures [58,75].

A critical gap in the DED-LB/M process is the lack of robust in-situ identification of defect types, sizes, and locations. This deficiency in real-time monitoring and control capabilities hinders the ability to act during the build process to prevent or mitigate defect formation, which is important for increasing the overall reliability of the DED-LB/M process. Therefore, understanding the mechanisms behind process-induced defect formation is vital for developing effective inspection methods and implementing corrective measures.

##### Cracking

**Figure 4:**
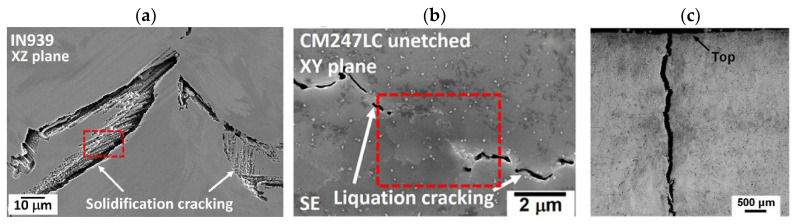
Major crack types observed in DED-LB/M fabricated structures made from nickel-based superalloys. The three primary crack types are: (**a**) Solidification cracking [89], (**b**) Liquation cracking [89], and (**c**) Cold cracking [90]. This figure is reprinted with permission of Elsevier [90].

Cracking is a common defect observed in structures fabricated using the DED-LB/M process. This defect can be generated both during and after the deposition process, occurring on the surface of the fabricated structures or within their internal layers. Generally, cracking in DED-LB/M fabricated structures is categorised into two main types: hot cracking and cold cracking [77]. Hot cracking, which includes solidification cracking and liquation cracking, occurs during the solidification process [19,78,79]. Solidification cracking occurs in the fusion zone of the deposit during the final stages of solidification [77,80], as illustrated in Figure 4a. This type of cracking is characterised by fractures along grain boundaries and is often associated with the presence of low-melting-point constituents that form liquid films between dendrites [73,81]. In contrast, liquation cracking generates in the heat-affected zone (HAZ) of previously deposited layers [47,79], as shown in Figure 4b It is caused by the partial melting of grain boundaries in the HAZ owing to the presence of low-melting-point constituents or segregates [82,83]. Importantly, once a liquation crack is generated, it remains in the HAZ of the as-deposited coating. With the deposition of subsequent layers, these remaining liquation cracks can grow larger layer by layer [79,84]. Figure 4c shows a micrograph of cold cracking, which primarily occurs after solidification, typically within hours or even days following the deposition process [77]. In contrast to hot cracking, this phenomenon takes place as the material approaches room temperature and is primarily driven by residual stresses generated during the rapid thermal cycling inherent to the DED-LB/M process [16]. These residual stresses result from repeated heating and cooling cycles, which cause thermal expansion and shrinkage, leading to the accumulation of internal stress within the applied material layers [85]. If these stresses are not adequately relieved during the cooling process, they can persist in the final structure, creating conditions conducive to crack formation [77,86]. Cold cracks often initiate at stress concentration points, such as microstructural inhomogeneities or pre-existing defects, and can propagate through regions of unfavourable microstructure, including areas with coarse grains or brittle phases [81]. The occurrence of cracks in the fabricated structures reduces their wear and corrosion resistance, significantly impacting the overall performance of the DED-LB/M fabricated structures [86,87,88].

##### Porosity

Porosity is a critical defect in materials, characterised by the presence of voids or empty spaces that significantly impact mechanical properties. In DED-LB/M fabricated structures, various types of porosity can arise, each with distinct formation mechanisms.

**Figure 5:**
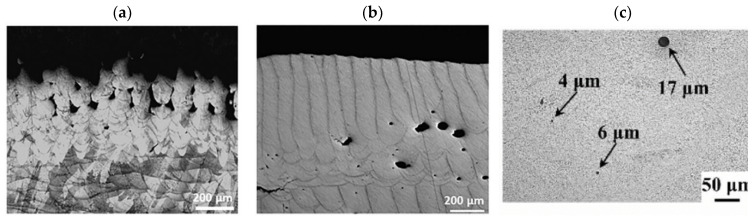
Major porosity types observed in DED-LB/M fabricated structures. (**a**) Lack of fusion (LoF) [103], (**b**) keyhole porosity [103], and (**c**) gas-induced porosity [104].

Lack of Fusion (LoF) porosity forms when inadequate energy density fails to fully melt and bond adjacent layers, resulting in irregular voids [91,92,93]. These defects range from 50 µm to several millimetres in size [94], as shown in Figure 5. In DED-LB/M, low input energy relative to scan speed and powder feed rate can leave unmelted powder particles in the processing zone, creating LoF defects. Additionally, inadequate heat source energy can lead to poor interlayer bonding [95,96]. LoF defects significantly compromise the mechanical properties, integrity, and performance of DED-LB/M fabricated structures by creating stress concentration points that can initiate cracks and reduce overall strength, ductility, and fatigue resistance [94].

Keyhole porosity, conversely, forms under conditions of excessive energy input, leading to large spherical pores due to material evaporation and unstable melt pool dynamics [97,98]. The formation mechanism of keyhole porosity involves a high-energy laser creating a deep cavity, or “keyhole,” in the molten material. This intense energy input vaporises metal, generating a recoil pressure that sustains the keyhole’s depth. If the keyhole becomes unstable due to fluctuations in the melt pool, the cavity may collapse, trapping gas bubbles that solidify as pores [99]. Keyhole pores are generally larger, ranging from 100 μm to several hundred micrometres in size, influenced by the process parameters and material properties [100].

Gas-induced porosity results from gas entrapment in the melt pool, often caused by high-velocity powder feedstock injecting gas bubbles during deposition [101]. These pores are typically spherical and generally smaller than keyhole pores, usually below 100 μm in diameter [99]. 

To mitigate porosity formation, it is essential to provide adequate energy to completely melt the metal powder while avoiding excessive heat input. This can be achieved through careful control of heat input during the DED-LB/M process, which involves optimising the balance between laser power, scan speed, and powder feed rate. Fine-tuning these parameters can significantly reduce the occurrence of both types of porosity, thereby improving the overall quality and integrity of DED-LB/M fabricated structures [94,102].

##### Inclusions

**Figure 6:**
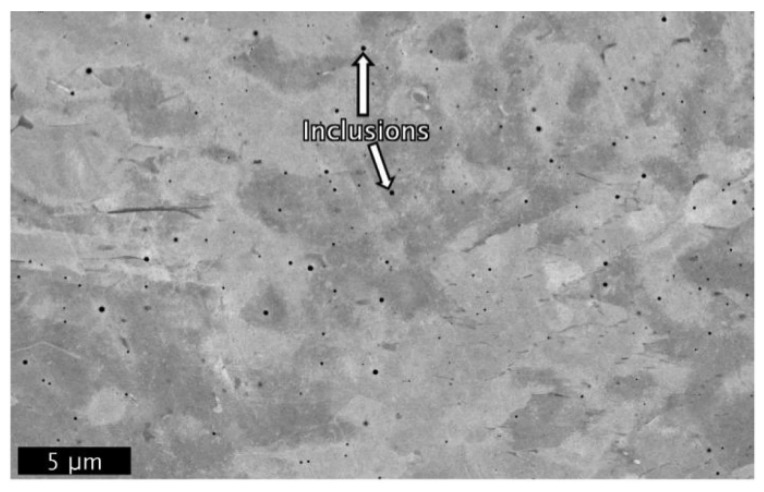
Inclusions observed in a DED-LB/M processed austenitic stainless-steel structure [105].

Inclusions are typically non-metallic particles embedded within the metal matrix, often characterised by a smooth, spherical morphology, as illustrated in Figure 6 [105]. These defects can occur in DED-LB/M fabricated structures, potentially impacting the mechanical properties and overall performance of the manufactured components [106]. In DED-LB/M fabricated structures, oxides and silicate inclusions have been detected, rich in elements such as Silicon (Si), Manganese (Mn), and Oxygen (O), with sizes varying between 0.05 to 15 μm [107]. The development of these inclusions is linked to the compositional micro segregation of deoxidiser and fluidity elements (e.g., Si, Al, and Mg) present in the powder feedstock [108]. The highly localised laser beam generates a high-temperature melt pool in the processing zone, creating conditions conducive to the formation of inclusions [109]. Under these elevated temperatures, oxygen molecules present in the powder, or the surrounding atmosphere react with oxide-forming elements in the powder feedstock [110]. Mitigating the formation of these inclusions is crucial for enhancing the quality and reliability of DED-LB/M fabricated structures. These inclusions can serve as focal points for stress concentration, potentially compromising the mechanical properties of the final structure.

### 3.2. In-Situ Process Monitoring Techniques in DED-LB/M Process

The quality assurance of structures fabricated through DED-LB/M processes has traditionally relied on iterative refinement of various process parameters and post-process evaluation methods. Manufacturers often rely on iterative adjustments to parameters such as laser power, powder feed rate, and scanning speed to optimise part quality [111]. This approach, while widespread, is time-consuming and often results in material waste and reduced efficiency.

Post-process evaluation methods, including microscopic examination of material structure, high-resolution surface analysis via electron microscopy, and non-invasive internal inspection using ultrasonic techniques, have been the primary means of assessing the quality of the DED-LB/M fabricated structures [27]. These analytical methods provide valuable insights into microstructure, potential defects, and overall part integrity. However, their delayed nature presents significant limitations in the DED-LB/M process.

The inherent drawback of these post-process evaluation methods is that defects or quality issues are only detected after part completion, potentially resulting in wasted materials, energy, and time. This post-process approach severely limits the ability to implement real-time defect detection and mitigation strategies to improve part quality during fabrication. Recognising these limitations, researchers are increasingly emphasising the development and implementation of in-situ monitoring techniques for defect identification during the DED-LB/M process. 

This section outlines a systematic overview of the in-situ monitoring technologies currently utilised to identify process defects during DED-LB/M fabrication and pinpoint their location in the fabricated structures. The focus is on three key aspects of these monitoring systems:

1. Process signature collection,
2. Feature extraction, and
3. Defect correlation.

The following monitoring methods will be critically examined:

4. Vision sensing
5. Thermal sensing
6. Spectral sensing and
7. Acoustic emission

*Process signature collection* involves an examination of the various sensors and data acquisition methods used to capture real-time information during the DED-LB/M process. *Feature extraction* discusses the techniques employed to extract meaningful features from the raw datasets collected during fabrication. *Defect correlation* explores the methods used to correlate the extracted features with specific defect types or quality issues. By conducting a thorough analysis of these aspects, a significant research gap was identified in process-induced defect detection and localisation within fabricated structures. This gap highlights the need for advanced techniques that not only identify defects but also provide accurate spatial information about their occurrence within the three-dimensional geometry of DED-LB/M fabricated structures.

#### 3.2.1. Vision Sensing 

Vision sensing has emerged as an important technique for identifying defects in real-time and monitoring process stability in the DED-LB/M process. This method employs optical cameras to capture instant images of the melt pool that forms through the interaction of the laser beam, powder feedstock, and substrate during the DED-LB/M process [11,23]. Researchers then apply advanced image processing algorithms to extract the melt pool’s geometrical characteristics from the captured images. Utilising these extracted features, researchers have developed a variety of data-driven methods to identify process-induced defects. These approaches utilise machine learning (ML) algorithms to establish correlations between melt pool characteristics and the occurrence of defects, enabling real-time quality control and process optimisation in DED-LB/M manufacturing [29,112].

##### Sensor Type 

Vision-based sensing in DED-LB/M primarily relies on non-contact high-speed imaging sensors, with charge-coupled device (CCD) and complementary metal oxide semiconductor (CMOS) cameras being the most prevalent [40,75]. These sensors are crucial for capturing real-time images of the melt pool during the DED-LB/M process, enabling in-situ monitoring and control [58]. However, the effectiveness of these sensors largely depends on their configuration within the DED-LB/M system.

Two main camera setup configurations have emerged in the literature: off-axis and coaxial [76]. Each configuration offers distinct advantages and limitations, significantly influencing the quality and type of data obtained during the DED-LB/M process.

The off-axis configuration, in which the camera is positioned at an inclined orientation relative to the laser beam and melt pool, has been commonly employed because of its simpler implementation and capacity to provide a side view of the process [40,113]. This configuration is particularly useful for observing layer deposition height and melt pool profile [76]. However, the oblique viewing angle introduces significant challenges in accurately measuring melt pool dimensions, necessitating complex image transformation and time-consuming calibration procedures. These limitations can potentially introduce errors in measurement and reduce the overall reliability of the monitoring system.

Conversely, the coaxial setup, where the camera shares the same optical path as the laser beam, offers superior visualisation of the melt pool’s horizontal dimensions, such as width, length, and area [114]. This top-down view provides more accurate measurements of these parameters compared to the off-axis setup. Nevertheless, the coaxial configuration is inherently limited to capturing only the top surface of the melt pool, making it impossible to obtain vertical dimensions like height and depth of the melt pool [75,114]. This limitation can be critical in applications where the melt pool’s vertical characteristics are essential for process control or defect detection.

To overcome the constraints of single-camera configurations, there has been some limited research employing dual-camera systems that combine both coaxial and off-axis configurations [115,116]. This approach aimed to provide complementary views of the melt pool and overall process dynamics, potentially offering a deeper and more holistic understanding of DED-LB/M operations. However, the implementation of such systems introduces additional complexity in terms of hardware integration and data synchronisation, which may limit their practical application in industrial settings.

Recent advancements in imaging techniques have focused on enhancing the clarity and quality of melt pool imaging. Strategies such as using laser illuminators [117], narrow bandpass filters [118], and specialised Near-Infrared (NIR) CMOS machine vision cameras with UV/VIS cut-off filters [119] have shown promise in reducing background interference and improving image quality. These enhancements enable more precise defect detection and process analysis. However, the effectiveness of these techniques in diverse DED-LB/M applications and materials remains to be fully explored. The advantages and disadvantages of these camera configurations and filtering techniques are summarised in Table 1, providing a clear comparison to aid selection based on specific DED-LB/M monitoring requirements.

While considerable advancements have been achieved in vision-based sensing for DED-LB/M, each sensor configuration presents its own set of trade-offs. The choice between off-axis and coaxial setups, or the implementation of a dual-camera system, should be carefully considered, guided by the demands of the DED-LB/M application, the process aspects that need to be monitored, and the practical constraints of system integration.

##### Process Signatures

The geometry of the melt pool has emerged as a critical quality indicator in DED-LB/M processes. Due to the complex thermal environment inherent in DED-LB/M, melt pool shapes exhibit significant variability, making manual categorisation of melt pool images both impractical and prone to inaccuracies [122]. To address this challenge, researchers have developed advanced image processing techniques aimed at extracting precise melt pool geometries from acquired images [116,117]. These methods are designed to overcome common imaging issues such as blurriness, glare, and noise, which often complicate the analysis of melt pool characteristics. These techniques have evolved beyond simple edge detection and thresholding methods to more sophisticated approaches that can handle complex imaging conditions in DED-LB/M processes. The image processing workflow typically involves several key steps: filtering to enhance image quality and reduce noise, threshold segmentation to isolate the melt pool from the background, edge detection to accurately outline melt pool boundaries, and feature extraction to determine geometrical characteristics such as width, length, and area [123,124]. 

Song et al.’s [123] phase congruency-based method represents a significant advancement over traditional intensity-based edge detection algorithms. By focusing on features that remain consistent across different scales and orientations, this technique demonstrates superior robustness in the presence of glare and blurriness, which are common issues in DED-LB/M imaging. This approach offered more reliable melt pool edge detection under challenging conditions, potentially improving the accuracy of process monitoring and control systems. However, the effectiveness of this method may be reduced in extremely low-contrast situations, limiting its applicability in certain DED-LB/M scenarios.

Sampson et al.’s [119] technique, that implemented the directional emittance phenomenon, addresses a fundamental limitation of traditional emissivity-based algorithms. By using optimised exposure times and analysing directional emission patterns, their method can distinguish between actual melt pool edges and surrounding areas more effectively. This is particularly valuable in DED-LB/M processes where emissivity values fluctuate during fabrication, potentially leading to measurement errors in conventional systems. However, this approach may require careful calibration for different materials and process parameters, which could limit its versatility across various DED-LB/M applications.

The enhanced edge detection technique introduced by Jinjin et al. [125], based on improved mathematical morphology, offers a promising approach to dealing with noisy imaging conditions. By combining noise reduction through morphological operations with specialised edge detection, this method achieves more reliable melt pool measurements in challenging environments. This could be especially beneficial in industrial DED-LB/M applications where image quality may be compromised by various factors. However, the morphological operations, while effective for noise reduction, could potentially alter fine details of the melt pool shape if not carefully tuned, potentially leading to inaccuracies in certain scenarios.

In recent years, researchers have leveraged ML algorithms to analyse captured melt pool images and extract the most relevant melt pool characteristics. A significant contribution to this area was made by Wang et al. [126], who developed an innovative physics-informed temporal convolutional network (TCN) method for predicting melt pool features in the DED-LB/M process. Their method demonstrated promising results in predicting key melt pool parameters, achieving an average percentage error of 4.6% for layer height and 3.4% for melt pool width and. A key strength of their approach lies in the integration of a comprehensive thermal model into their ML framework. By generating a dataset of peak melt pool temperatures as input for the TCN, the researchers effectively combined physics-based modelling with ML techniques, potentially enhancing prediction accuracy beyond purely data-driven approaches. However, the performance of this method in an industrial setting, where process parameters may be more variable, remains to be validated. Mi et al. [117] introduced a deep convolutional neural network (deep-CNN) architecture for extracting horizontal melt pool characteristics (width, length, and area) from melt pool and spatter images in the DED-LB/M process. Their approach utilised multiple lightweight architectures to reduce detection time while improving accuracy through an enhanced penalty function. The model achieved 94.71% accuracy in extracting these three key melt pool characteristics. However, the computational requirements of deep-CNN architectures may pose challenges for real-time processing in industrial DED-LB/M applications.

While these advancements in melt pool characterisation are significant, a key challenge remains, how to accurately utilise these melt pool characteristics to correlate with process-induced defects.

##### Analysis of Melt Pool Features to Predict Defects

A comprehensive analysis of the techniques presented in Table 2 reveals a significant correlation between the geometric characteristics of the melt pool, such as size and shape, and the quality of structures fabricated using the DED-LB/M process. The studies indicate a balanced use of co-axial and off-axis camera setups in vision-based defect detection. Most research has focused on extracting geometric characteristics of the melt pool as key features in defect prediction, with CNN-based approaches consistently achieving high accuracy rates exceeding 90%. Especially, porosity emerged as the most detected defect, highlighting its prevalence in DED-LB/M processes. A key observation was the association between irregular melt pool shapes and areas containing defects, contrasting with the uniform melt pools typically observed in high-quality, defect-free regions. [127].

Researchers have employed various ML techniques to establish relationships between coaxially captured melt pool images and the quality of fabricated structures in the DED-LB/M process. For instance, Kao et al. [128] employed multiple ML techniques to correlate geometric features extracted from melt pool images with fabrication quality. Their methodology utilised autoencoders and convolutional neural networks (CNNs) to extract the melt pool width from the images, while also measuring the height of the melt pool and calculating the ratio of the height of the HAZs to the deposition height using a CCD camera on cross-sectioned samples. The extracted features served as inputs for four classification models: softMax neural network (SNN), support vector machine (SVM), random forest (RF), and linear regression (LR), all of which achieved accuracy rates exceeding 95%. A significant drawback of their approach is the reliance on traditional cross-sectioning techniques for evaluating fabrication quality, which is cumbersome and labour-intensive. Additionally, the study did not classify specific defect types, such as porosity or cracks, limiting its applicability for comprehensive defect detection. 

Recent advancements in ML algorithms have further enabled researchers to identify correlations between extracted melt pool features and process-induced defect types in DED-LB/M processes. Researchers have observed that both horizontal characteristics, including melt pool width, length, and area, and vertical characteristics, such as melt pool depth and layer height, are the most relevant input features for ML classification models aimed at identifying potential defects in fabricated structures [126,129,130,131]. This progression highlights the significance of integrating advanced analytical techniques to enhance defect detection capabilities.

The literature highlights various successful applications of ML algorithms in identifying defects such as lack-of-fusion [132], spatters [117], and geometric distortions [133] by utilising melt pool characteristics as inputs for classifiers. Montazeri [132] et al. demonstrated a notable application of dual sensor fusion by combining a spectrometer with an optical CCD camera for melt pool observation in the DED-LB/M process. The researchers extracted three key signal features: two line-to-continuum ratio signatures from the spectrometer and the melt pool area from a CCD camera. By employing these features in a graph-theoretic supervised ML approach, they achieved an accuracy of 85% for a two-level classification of lack-of-fusion defects.

Building upon this approach, several researchers have further explored the potential of vision-based melt pool features to identify porosity in DED-LB/M fabricated structures [116,134,135,136,137,138]. Shin et al. [116] utilised advanced image preprocessing techniques to extract essential melt pool features from captured images, including size (area, width, height) and shape. These statistical features were then used as inputs for multiple binary classification models based on ML algorithms, specifically K-Nearest Neighbours (KNN), SVM, and ANN. The developed system demonstrated exceptional performance in identifying porosity in DED-LB/M fabricated structures, with the SVM achieved the highest average accuracy of 92.7% in defect classification. Yin et al. [137] developed an innovative method for identifying localised porosity in DED-LB/M fabricated structures using a dynamic mapping strategy and a Multibranch Fusion Convolutional Neural Network (MBFCNN). The method employs a coaxially integrated camera to capture real-time melt pool images and constructs experiment-based datasets using X-ray CT for localised porosity detection. The MBFCNN model, consisting of multi-branch feature extraction, feature fusion, and decision-making modules, establishes a precise correlation between in-process images and localised porosity. Validation results demonstrated the MBFCNN’s superior performance over traditional single-branch CNNs in accurately predicting porosity defects in DED-LB/M fabricated structures. However, this method requires substantial high-quality training data and is computationally intensive, making real-time application challenging.

Moreover, studies by Zhang et al. [135] and Pandiyan et al. [134] showcase high classification accuracies for porosity detection using CNNs and self-supervised learning algorithms. Despite these advancements, a significant limitation remains the reliance on batch-learning approaches in ML models for defect detection. The transient thermo-physical behaviour of the melt pool is inherently volatile and can vary significantly with changes in process parameters. This variability can lead to considerable differences in the geometrical characteristics of the melt pool, potentially failing these algorithms to identify defects or anomalies that were not present in the initial training data.

Vision-based defect detection systems have shown promise in identifying porosity and other flaws, but there remains a significant research gap in correlating melt pool characteristics observed through vision systems with the formation of process-induced cracks in DED-LB/M fabricated structures. While many studies have investigated ML techniques to detect process-induced defects, they predominantly utilise offline training methods for model development. The practical aspects of deploying these models in active manufacturing settings, particularly the need for rapid data processing and decision-making, remain largely unexplored.

**Table 2:** Summary of vision-based defect detection approaches in the DED-LB/M process.

Process	Devices Used	Extracted Features (Input)	ML Algorithms	Defect Detection	Performance indicators	Ref.
DED-LB/M	CCD Camera (Off-Axis) Spectrometer (Off-Axis)	Melt pool plume area, line-to-continuum ratio signatures	SVM	Lack-of-fusion	F-score—85%	Montazeri et al. [132]
	CCD Camera (Off-Axis)	Geometric characteristics of melt pool (e.g., area, length, width, etc.).	D-CNN	Spatters	Accuracy—95%	Mi et al. [117]
	High-speed digital camera (Co-axial)	Geometric characteristics of melt pool (e.g., area, length, width, etc.)	CNN	Porosity	Accuracy—91.2%	Zhang et al. [139]
	CCD Camera (Co-axial)	Geometric characteristics of melt pool (e.g., area, length, width, etc.)	RF, SVM, k-NN	Porosity	Accuracy—97%	Pandiyan et al. [134]
	CCD Camera (Off-Axis) Pyrometer (Off-Axis)	Melt pool size (area, width, height), and temperature	KNN, SVM, ANN	Porosity, Melting balls	Avg. Accuracy—92.7%	Shin et al. [116]
	CMOS Camera (Co-axial) X-ray	Geometric characteristics of melt pool (e.g., area, length, width, etc.)	MBFCNN	Porosity	Accuracy—90.1 %	Yin et al. [137]
	CMOS Camera (Off-Axis)	Geometric characteristics of melt pool (e.g., area, length, width, etc.)	CNN	Porosity	Accuracy—99%	Li et al. [138]

##### Current Challenges, Existing Limitations, and Paths for Future Exploration

Vision-based sensing systems for DED-LB/M processes have made significant progress in monitoring melt pool dynamics and detecting surface defects. However, these systems face several critical limitations that hinder their effectiveness in comprehensive defect detection:

1. Vision-based coaxially mounted optical cameras are good at capturing the horizontal dimensions of a melt pool, such as its width, length, and area. However, off-axis cameras are needed to measure the vertical dimensions, like the height and depth. Using multiple camera systems for comprehensive melt pool monitoring is difficult and expensive due to the combination of different hardware and software requirements. Therefore, future research should focus on finding ways to reduce system complexity and cost while still providing accurate melt pool characterisation.
2. Another most significant challenge is the inability of vision-based systems to identify subsurface flaws, particularly cracks that may form due to the complex thermal gradients and residual stress patterns inherent to the DED-LB/M process. Therefore, this method may not provide a complete picture of defect formation and propagation throughout the build process. Consequently, it requires a combination with other complementary techniques like X-ray imaging to provide a more comprehensive view of both surface and subsurface defects.
3. The high-temperature environment and intense light emissions during the DED-LB/M process can interfere with image quality, potentially leading to inaccurate defect detection. Further investigations are required to design more resilient imaging systems that can operate reliably under these extreme conditions.
4. Many current systems struggle with real-time data processing and decision-making. The computational demands of processing high-resolution melt pool images in real-time and the potential latency in defect prediction could hinder immediate responsiveness for in-situ process control. Developing more efficient algorithms and hardware solutions for rapid data processing is crucial.
5. Many ML models utilised for defect identification in DED-LB/M processes rely on batch-learning approaches. These approaches require complete datasets from entire DED-LB/M build processes for model development and training. However, these models struggle to adapt quickly to new or unseen data, especially when dealing with the dynamic and complex heat transfer conditions in DED-LB/M processes. The transient thermo-physical behaviour of the melt pool is highly volatile and can vary significantly between structures, making it difficult for the models to adjust to changing conditions or detect new defects during production. As a result, these models may fail to identify defects or anomalies present in the initial training data, potentially leading to quality issues in manufactured structures. To overcome these limitations, there is a need for more advanced, adaptive ML models capable of continuous learning and updating during the DED-LB/M process, providing real-time defect detection capabilities.

#### 3.2.2. Thermal Sensing

Thermal sensing is an important monitoring technique used in DED-LB/M processes, offering significant insights into the dynamics of the melt pool and its thermal history. The main features obtained from thermal sensing data include the geometrical characteristics of the melt pool, such as area, length, and width, as well as its temperature distribution [75]. These thermal characteristics are necessary for understanding the thermal and physical history of the fabricated structures, as they can aid in predicting potential defects that may arise from irregularities in melt pool behaviour and heat transfer [76,114]. Various ML algorithms are commonly applied to analyse the extracted thermal features. These algorithms can be trained to identify correlations between thermal patterns and defect formation, enabling real-time quality control and process optimisation [29,112]. Thermal sensing offers advantages such as providing real-time information on melt pool thermal characteristics and detecting process-induced defects.

##### Sensor Type

A variety of thermal sensors were utilised to capture the temperature distribution of the melt pool during the DED-LB/M process, with infrared (IR) thermal cameras and pyrometers being among the most used devices. These sensors were typically configured in either off-axis or coaxial setups [58,140]. IR cameras were particularly useful for quantitatively investigating melt pool morphology, as each thermal image reflects the temperature distribution across the melt pool area. The complex thermal distribution in DED-LB/M processes results in varying melt pool shapes, which can be analysed to identify the process stability and structural quality [40,58].

Herzog et al. [121] developed an advanced setup utilising three strategically positioned IR thermal cameras along various optical axes to observe comprehensive melt pool shape and vertical displacement during the entire fabrication process. While this approach enhances the understanding of melt pool dynamics, it may be limited by the complexity of interpreting data from multiple sensors, which can introduce challenges in correlating results across different camera angles. Kim et al. [141] developed an innovative real-time layer height estimation system by employing a coaxially mounted IR thermal camera with 1200–2500 K calibrated temperature range. Taking a dual-perspective approach, Sun et al. [115] combined both off-axis and coaxially installed IR thermal cameras to monitor melt pool morphology characteristics during DED-LB/M. This integrated setup provided a more complete view of the melt pool dynamics, allowing for better correlation between thermal patterns and potential defects or process instabilities. However, the reliance on multiple sensor types can complicate data integration and analysis, potentially leading to inconsistencies in the interpretation of melt pool behaviour.

To accurately determine melt pool temperatures using IR thermal cameras, proper calibration is important, as the emissivity of the feedstock material must be known in advance for precise temperature measurements [142]. However, this property is complex, as it depends not only on material composition but also on surface roughness and temperature, particularly for metals [58]. In DED-LB/M processes, rapid phase transformations pose additional challenges for accurate temperature measurement due to difficulties in maintaining consistent emissivity estimates throughout the build process.

To address these limitations, researchers have increasingly adopted a dual-sensor approach, combining two-colour pyrometers with thermal cameras [58]. Two-colour pyrometers offer several advantages, including emissivity independence, improved accuracy in environments with varying or unknown emissivity, a wider measurable temperature range, and the ability to provide real-time measurements. By combining thermal cameras with two-colour pyrometers, researchers can obtain a more thorough and accurate understanding of the thermal behaviour in DED-LB/M processes, leading to enhanced process control and defect detection capabilities. 

Li et al. [143] measured the temperature and area of the melt pool using a combination of a camera and a pyrometer. Subsequently, they mapped these thermal features into three dimensions to gain deeper insights into the process dynamics. Additionally, Ouidadi et al. [144] utilised a thermal camera to capture high-resolution images of the melt pool and employed a pyrometer to accurately measure its temperature. This dual approach enabled them to develop a classification system that effectively categorised melt pool conditions as normal or defective.

However, traditional melt pool monitoring methods face challenges when applied to detecting anomalies on a layer-by-layer basis. The thermal distribution varies continuously across different layers within the same build, making it difficult to establish a single benchmark distribution applicable to all layers. Furthermore, thermal behaviour can fluctuate significantly for complex components based on the specific printing path, which must be considered in anomaly detection strategies [142]. 

To overcome these challenges, researchers have proposed innovative in-situ methods for detecting anomalies at each layer of the build. These methods analyse the shape changes of melt pools and HAZs as the process progresses, offering deeper insights into thermal behaviour throughout the fabrication process [145]. Additionally, researchers have employed infrared cameras to monitor the entire thermal field generated during fabrication, identifying regions with elevated temperatures and significant fluctuations in thermal gradient [146]. This broader perspective allows for a more nuanced analysis of the process, potentially revealing areas prone to defect formation or process instabilities.

##### Process Signatures

The literature on melt pool feature extraction in DED-LB/M processes reveals several advanced methods integrating image processing with ML techniques for defect detection. The key features extracted typically include geometric characteristics such as melt pool width, length, and area and thermal features including mean and maximum temperatures, cooling rates, and thermal gradients. Among the various approaches, traditional image processing techniques and sophisticated ML models show promise but also limitations. Techniques like Generative Adversarial Networks (GANs), including image-enhancement GANs (IEGANs), improve thermal image quality by enhancing contrast, which supports better segmentation and feature extraction [122].

More specialised methods, such as the spherical transformation technique used by Khanzadeh et al. [147], address the challenge of handling three-dimensional thermal data. By transforming melt pool data into a spherical domain, this method standardises varying melt pool shapes, facilitating the estimation of missing data points. While this approach is well-suited for complex thermal analysis, it may be limited in cases where melt pool symmetry does not align with a spherical model, potentially reducing its general applicability across different DED-LB/M conditions.

In another approach, Ouidadi et al. [144] implemented a two-step preprocessing approach using histogram of oriented gradients (HOG) features and K-means clustering to classify melt pool images as normal or defective. Although effective for binary classification, the reliance on cropped image regions around peak temperature points could overlook significant information outside these focal areas, potentially limiting classification accuracy when temperature gradients are broader.

Ye et al. [148] further explored ML algorithms to correlate melt pool width, area, and thermal metrics with melt pool depth, height, and dilution. This integration of thermal features with ML provides a deeper insight into the melt pool dynamics. However, while this method demonstrates high accuracy, it does not fully address the variability in melt pool behaviour due to changing process parameters, which may result in unreliable predictions for defects under different conditions.

Overall, while these techniques demonstrate advancements in melt pool analysis, they share common limitations in their adaptability to real-time conditions and dependence on high-quality datasets. The main challenge moving forward is to effectively translate these sophisticated thermal features into consistent, reliable predictors of defect formation.

##### Analysis of Thermal Features to Predict Defects

The analysis of thermal features in DED-LB/M processes has garnered significant attention, with researchers implementing various statistical and ML algorithms to establish correlations between extracted thermal features and process-induced defects [121,144,145,146,149,150,151,152,153], defective areas [154], and layer height estimation [141]. Examining Table 3 revealed that a diverse array of devices, including IR thermal cameras and pyrometers, were employed to capture morphological features of melt pools and their temperature distributions. The results indicated that ML methods utilising these thermal features achieved high accuracy rates in detecting porosity, with overall accuracy exceeding 96%. This overview underscored the strengths of different approaches while also highlighting the necessity for a critical evaluation of the methodologies employed and their inherent limitations.

D’Accardi [150] et al. refined the correlations between process parameters and extracted thermal features through comprehensive statistical analyses. They found that the mean apparent temperature exhibited the strongest correlation with process-induced defects. However, this research lacked specificity regarding the types of defects being analysed, failing to distinguish between issues such as cracks and porosity.

Mazzarisi et al. [146] addressed this gap by monitoring the entire thermal field during the multitrack DED-LB/M process. Their investigation revealed numerous cracks in regions with elevated temperatures and significant thermal gradient variations. Similarly, Wu et al. [149] observed changes in peak temperature when the laser beam traversed simulated cracks on DED-LB/M fabricated structures. These studies collectively indicate a strong potential for using thermal features to predict crack formation; however, a comprehensive correlation among various thermal features and process-induced cracks remains underexplored.

In addition to crack detection, researchers have investigated multi-sensor systems for porosity detection in DED-LB/M fabricated structures. Herzog et al. [121] developed a custom data processing algorithm using MATLAB (2022a) to track dynamic changes in melt pool position, morphology, and dimensions based on input from a three-sensor array. Their results demonstrated effectiveness in detecting process-induced porosity, particularly in challenging geometries like thin-walled structures. However, it is important to note that while real-time data collection was performed, processing occurred offline, which may limit the system’s responsiveness to anomalies during fabrication.

Khanzadeh et al. [147,153] conducted two significant studies on porosity prediction in DED-LB/M fabricated structures. In the first study, an innovative approach was introduced utilising melt pool morphological characteristics extracted through functional principal component analysis (FPCA) to predict porosity in as-built structures. Five supervised ML algorithms were compared, with KNN achieving an impressive accuracy of 98.44% in classifying melt pools. While this high accuracy is noteworthy, it raises questions about the generalisability of these ML methods across different materials and process conditions. In the subsequent study, the methodology was advanced by incorporating thermal distribution analysis of the melt pool. This approach employed Self-Organising Maps (SOMs) to analyse two-dimensional melt pool image streams and predict porosity locations with approximately 96% accuracy when using an appropriate SOM model with thermal profile data. Validation of findings using X-ray tomography on a thin-wall specimen supports the robustness of this approach but underscores the need for further exploration into how these methods perform under varying operational conditions.

Bappy et al. [145] introduced a novel approach for real-time layer-wise porosity detection in DED-LB/M fabricated structures. This method utilised temperature-based segmentation of thermal images to differentiate between melt pools and their associated HAZs. From these segmented regions, several new layer-wise key process features were extracted, which served as input for a SVM framework, enabling automated layer-wise anomaly detection. The SVM model was trained to distinguish between normal and anomalous thermal patterns, potentially indicating porosity. While the proposed model achieved an impressive anomaly detection accuracy of 96%, it is crucial to consider whether this accuracy can be maintained across diverse geometries or material types.

Researchers have increasingly utilised ML-based data fusion methods to predict porosity in DED-LB/M fabricated structures by integrating melt pool thermal history data from multiple sensors and thermal simulations. For instance, Gaikwad et al. [152] developed a method that combines thermal simulations, in-situ sensing, and ML techniques for anomaly detection. They extracted statistical features from in-situ melt pool temperature measurements using a two-colour pyrometer. While the study achieved a notable prediction accuracy improvement from 80% to 90% by combining data sources, concerns remain regarding the model’s robustness across varying conditions, which could limit its practical application.

To analyse melt pool thermal behaviour, Tian et al. [151] introduced a method that synthesise information from pyrometry and sequential IR thermal imaging. They created two specialised neural networks: PyroNet for correlating pyrometry images with layer-wise porosity and IRNet for correlating sequential infrared images. This combined approach achieved over 96% accuracy; however, like many ML models in DED-LB/M processes, it relies on batch-learning methods that struggle to adapt to new or unseen data due to the dynamic heat transfer conditions inherent in DED-LB/M.

To address these adaptability challenges, Ouidadi et al. [144] proposed continual online learning versions of SOMs and K-means models. Tested on a thin wall dataset, these models showed improved performance in detecting anomalous melt pool images, achieving 76% accuracy for K-means and 97% for SOM. While these findings underscore the promise of continuous adaptive learning techniques for real-time defect recognition, further validation across various operational conditions is crucial to ensure reliability.

Overall, despite substantial progress in utilising ML-based methods for porosity detection in DED-LB/M processes, a notable research gap persists regarding the establishment of strong correlations between thermal characteristics and process-induced cracks.

##### Current Challenges, Existing Limitations, and Paths for Future Exploration

Thermal-based sensing techniques have gained prominence as reliable approaches for real-time defect identification and process monitoring in DED-LB/M processes, offering valuable insights into melt pool dynamics and structure quality. However, several challenges and constraints remain, hindering the broad implementation and performance of these techniques in industrial applications.

1. Implementing multiple sensors, such as thermal cameras and pyrometers, in industrial settings can be complex and costly. The installation of these diverse sensors may disrupt existing production workflows and require significant modifications to manufacturing setups. Future research should focus on creating compact, multi-sensor units that can be retrofitted to existing DED machines with minimal disruption to production workflows. Additionally, efforts should be made to standardise sensor interfaces and data formats to facilitate easier integration across different DED-LB/M systems and manufacturers.
2. Accurate temperature measurement remains a persistent challenge in DED-LB/M owing to the inherent process variability and the diverse thermal behaviours of different materials. Emissivity calibration is particularly crucial for reliable thermal readings but can be difficult to achieve consistently across different materials and surface conditions. Research into adaptive calibration methods that can account for changing emissivity and surface conditions during the DED-LB/M process could improve temperature measurement accuracy.
3. While thermal sensing has shown promise in detecting porosity, there’s a significant gap in reliably correlating thermal characteristics with interlayer crack formation and propagation. The ability to differentiate between defects such as cracks and porosity based on their thermal signatures is an area that requires further investigation. Further investigation into the unique thermal signatures of different types of defects could lead to improved differentiation between cracks, porosity, and other flaws.
4. The need for complete datasets often limits Traditional ML models’ ability to detect defects in DED-LB/M fabricated structures and makes them struggle with new or dynamic conditions. Exploring continual online learning, as shown by Ouidadi et al. [144], could significantly improve these models’ adaptability for real-time monitoring and defects identification in DED-LB/M processes.

#### 3.2.3. Spectral Sensing

While vision systems provide spatial information and thermal sensing captures temperature distributions, spectral sensing delves into the atomic and molecular level interactions occurring during material deposition [156]. This complementary approach enhances the ability to detect and characterise process-induced defects with greater precision in DED-LB/M fabrication.

Spectral sensing has emerged as a powerful technique for detecting process-induced defects in DED-LB/M fabricated structures. This method employs spectrometers to capture and analyse the emission spectra generated during the deposition process [157]. By monitoring variations in spectral signatures, researchers can identify and characterise defects in real-time.

Plasma emission spectroscopy (PES) and optical emission spectroscopy (OES) have emerged as the most effective spectral analysis methods for defect detection in the DED-LB/M process [158,159,160,161]. PES represents a specialised application, focusing on the analysis of light emitted by plasma formations during high-energy manufacturing processes [29]. In the DED-LB/M process, PES exploits the unique spectral fingerprints produced when a high-energy laser interacts with powder feedstock. This interaction creates a localised high-temperature zone, partially vaporising and ionising the metal to form a plasma plume above the melt pool. As excited atoms and ions in this plasma return to lower energy states, they emit photons with characteristic wavelengths. The resulting emission spectra provide crucial information about elemental composition, plasma temperature, and process conditions [162]. OES, while similar in principle to PES, analyses the light emitted from the process zone but focuses on the overall optical spectrum rather than just plasma emissions [163]. This technique can capture spectral information from the melt pool, surrounding heated material, and any vapor formations, providing a more comprehensive view of the deposition process.

By capturing and interpreting these spectra, researchers can gain deep insights into material properties, process stability, and potential defect formation, making PES an invaluable tool for in-situ monitoring and quality assurance in the DED-LB/M process.

##### Sensor Type 

Spectral sensing in DED-LB/M processes typically relies on two primary types of equipment: spectrometers and fibre optic probes. Spectrometers are the cornerstone of spectral analysis which capture the light emitted from the plasma plume during deposition and separate it into its constituent wavelengths, enabling detailed analysis of elemental composition and plasma conditions [161,164,165]. Complementing the spectrometers, fibre optic probes play a crucial role in light collection. Collimating lenses are commonly used to gather light from the plasma plume and direct it into the spectrometer. Multi-channel fibre optic cables are also utilised, allowing for simultaneous measurement at multiple points or angles around the deposition area [158,160]. This combination of spectrometers and fibre optic probes forms the backbone of most spectral sensing setups in the DED-LB/M process. 

##### Process Signatures

After acquiring radiation data using spectrometers during the DED-LB/M process, researchers employ various feature extraction techniques to identify the most relevant spectral features that correlate with process anomalies. This critical step involves analysing radiation intensity, creating time-domain diagrams of specific wavelengths, and applying statistical methods to characterise intensity fluctuations [163]. Common features extracted include line intensities of specific elements, standard deviations of spectral lines, plasma temperature, and electron density [164].

Time-domain analysis is frequently utilised to reveal temporal patterns related to defect formation, while statistical methods such as Statistical Process Control (SPC) were applied to analyse intensity fluctuations and correlate them with forming defects [164]. More advanced techniques involve ML approaches, such as RF classifiers, to identify important spectral features for defect recognition. For instance, Mazumder et al. [165] found that intensities at specific wavelengths (414.234 nm for Fe I and 396.054 nm for Al I) and kurtosis of spectra in certain wavelength ranges were effective for porosity recognition.

Some researchers have proposed using spectral normalisation and correlation techniques to simplify computational requirements [132,166]. Additionally, unsupervised learning methods, such as Long Short-Term Memory (LSTM) based autoencoders, have been employed to automatically extract features from raw spectral data, capturing both auto- and cross-correlations in the time-series data [160].

These diverse feature extraction methods enable researchers to identify key spectral signatures associated with different process anomalies. By applying these techniques, researchers can develop more robust and accurate systems for detecting and classifying defects in DED-LB/M fabricated structures.

##### Analysis of Spectra Features to Predict Defects

Table 4 summarises various spectral analysis techniques and ML algorithms utilised to establish correlations between extracted spectral features and process-induced defects. The table highlights a range of approaches employing spectrometers to capture critical spectral features, including emission energy and line-to-continuum ratios. These techniques have demonstrated varying performance indicators, with some methods achieving good accuracy in detecting porosity and other defects.

Valdiande et al. [161] developed a quality monitoring system for DED-LB/M using plasma spectroscopy, background radiation analysis, and spectral correlation techniques. Their research showed that the average intensity emitted by the plasma, known as plasma RMS, could effectively detect changes in process conditions like gas or powder flow rates without needing to identify specific spectral emission lines. However, this approach lacks the specificity needed to identify and classify specific types of anomalies (e.g., cracks or porosity). 

Mazumder et al. [165] explored the integration of PES with ML algorithms for real-time porosity identification in DED-LB/M fabricated structures. They collected time- and position-synchronised spectra during the DED-LB/M process and correlated these with X-ray CT scans to assess the quality of fabricated structures. By extracting 18 features from the spectra and training a RF classifier, they achieved a classification precision of 83% for detecting porosity. However, their method’s accuracy decreased for higher layers due to variations in deposition thickness, indicating a limitation in generalisability across different build conditions. The researchers emphasised the need for strict control of deposition thickness when using spectroscopy, highlighting a significant challenge in achieving consistent results.

Their subsequent research introduced an unsupervised ML approach utilising a LSTM based autoencoder coupled with K-means clustering. This approach automatically identified features in time-series spectral data without requiring extensive labelled data or deep knowledge of plasma-material interactions. This approach showed promising results in real-time porosity identification, with high accuracy in reconstructing input spectra and classifying build quality under different conditions. This technique could reduce the dependency on labelled training data, but its effectiveness may still rely on the quality of spectral data and the consistency of the manufacturing environment [160]. 

Wasmer et al. [158] combined AE and OES with ML techniques to monitor and classify process regimes in DED process. The study examined four different chemical compositions and two sets of process parameters, leading to the identification of various process regimes, including conduction mode and varying levels of LoF defects. For feature extraction, raw acoustic signals were processed to obtain time and frequency domain features, while OES utilised intensity peaks corresponding to the emission spectra. Seven ML algorithms were employed to classify the chemical compositions and process regimes. The findings revealed that OES significantly outperformed AE in classification accuracy, with OES achieving accuracies between 78.25% and 96.38%, compared to AE’s range of 57.79% to 78.89%. The lower accuracy of the AE-based classification was attributed to very high noise content in the raw acoustic signal, highlighting the need for advanced signal processing algorithms to improve AE-based classification in the challenging DED-LB/M process environment.

In summary, the integration of spectral analysis techniques with ML has shown significant promise in monitoring process conditions and detecting defects in DED-LB/M processes. While advancements have been made in identifying porosity and understanding thermal features, challenges remain in achieving specificity and generalisability for various defect types, such as cracks. Continued research is essential to refine these techniques and enhance their applicability in real-time quality control within DED-LB/M manufacturing.

##### Current Challenges, Existing Limitations, and Paths for Future Exploration

While spectral sensing techniques have shown significant promise for real-time defect identification and process monitoring in DED-LB/M processes, several challenges and limitations persist:

1. The acquisition of spectral data is strongly influenced by the sensor’s relative placement and the thickness of the deposited material. This sensitivity can lead to inconsistent results, especially in DED-LB/M systems where deposition thickness may vary. Further investigations are required to design more resilient sensing strategies capable of accommodating changes in both deposition thickness and sensor placement.
2. Some materials may not emit strong line emissions, resulting in a low signal-to-noise ratio, particularly when operating under low laser power conditions. This limitation can affect the accuracy and reliability of spectral analysis for certain materials. Further investigation into enhancing signal detection and processing for a wider range of materials is needed.
3. While some approaches, like those proposed by Valdiande et al. [161], aim to simplify computational requirements, many spectral analysis techniques still require significant processing power. This can be a challenge for real-time monitoring in industrial settings. Future work should focus on developing more efficient algorithms and hardware solutions to enable real-time processing of spectral data.
4. Current spectral analysis techniques often struggle to classify specific types of defects based on their spectral signatures. While some research has successfully correlated spectral data with porosity, as demonstrated by Mazumder et al. [160] who reported a classification precision of 83% for detecting porosity, the detection of interlayer crack formation using spectral sensing remains largely unexplored. To date, no significant studies have successfully correlated spectral signatures specifically with crack formation in DED-LB/M processes. The ability to differentiate between defects, for instance cracks and porosity, based on their spectral signatures is an area that requires substantial further investigation.

#### 3.2.4. Acoustic Emission

Acoustic emission (AE) monitoring technique has emerged as an effective approach for identifying defects during the DED-LB/M process. This method analyses acoustic signals produced by laser-material interactions, offering valuable information on the development of both surface and sub-surface defect formation. AE is regarded as a viable approach to address the constraints of camera-based methods in detecting internal flaws during the DED-LB/M process [27,40]. 

During DED-LB/M, complex physicochemical processes occur as the high-energy laser beam interacts with the metal powder. These processes generate a series of acoustic vibrations, which manifest as AE signals. These signals carry valuable information about various structural anomalies, including cracks and pores, within the material as it is being deposited and solidified. By capturing and analysing these acoustic signals, researchers can obtain valuable insights into the material’s internal characteristics and potential defects as they develop during the DED-LB/M process [114]. The ability of AE to continuously monitor the entire build process enables prompt defect detection, potentially allowing for immediate adjustments to manufacturing parameters. This real-time information is invaluable for monitoring and potentially controlling the quality of fabricated components.

##### Sensor Type

Table 5 illustrates the various acoustic sensors utilised for in situ monitoring in the DED-LB/M process, highlighting their device types and operational frequency ranges. Acoustic monitoring systems in DED-LB/M processes typically employ two main categories of sensors:

1. Structure-borne acoustic sensor and
2. Airborne acoustic sensor.

Structure-borne acoustic sensors are directly attached to or integrated with the substrate during the DED-LB/M process. This configuration allows these sensors to detect vibrations and elastic waves that propagate through the solid material because of laser interactions with both the material and the substrate. Following amplification by a preamplifier, the acquired signals are recorded by a DAQ system. Typically, structure-borne acoustic sensors operate within a frequency range of 1.0 kHz to 1.0 MHz.

Numerous investigations have highlighted the effectiveness of structure-borne sensors in capturing signals associated with defects during the DED-LB/M process [167,168,169,170]. The literature indicates that wide-band piezoelectric sensors are typically mounted on the underside of the substrate to acquire acoustic signals during the DED-LB/M process [167,170]. To enhance the transmission of weak elastic waves, a coupling agent is often applied between the sensor and the substrate. This configuration improves signal quality, allowing for more accurate detection of defects. Additionally, these sensors can be affixed directly to the substrate of the part being built to monitor acoustic emissions throughout the DED-LB/M process [168,169].

Structure-borne sensors face considerable challenges in DED-LB/M operations, as the elevated process temperatures may compromise their integrity when mounted directly onto the workpiece. To mitigate this issue, some researchers have developed cooling systems to safeguard these sensors from excessive temperatures. For example, Kaiqiang et al. [170] employed double-sided contact circulating water cooling to maintain the temperature of the AE receiver’s installation base below its tolerance threshold of 200 °C. This cooling system minimises signal loss and attenuation during transmission under high-temperature conditions. Despite these thermal management solutions, structure-borne sensors still present limitations in terms of flexibility and adaptability. Their fixed positioning, necessitated by direct contact with the workpiece, restricts their ability to capture signals from different areas of complex geometries or large-scale builds. In contrast, airborne acoustic sensors offer greater versatility as they can be positioned at a distance from the build area, allowing for more dynamic monitoring configurations to capture process-related acoustic signatures. 

Airborne acoustic sensors, including condenser, pre-polarised, and optical microphones, have been widely utilised in prior research to record acoustic emissions during the DED-LB/M process. These sensors are versatile, typically operating within a frequency range of 1 kHz to 1 MHz, as illustrated in Table 5. They have been utilised to capture a diverse array of acoustic signatures related to different aspects of DED-LB/M, such as process stability, material flow, and defect formation. 

The literature reports varying placements of microphones in relation to the laser processing zone and differences in data acquisition rates for capturing acoustic signatures during the DED-LB/M process. In one experimental configuration, a microphone was placed 6 cm from the laser focal point, acquiring signals with a 51.2 kHz sampling rate and covering the frequency band of 6.3–20 kHz [177]. In another configuration, a pre-polarised microphone was placed approximately 10 cm from the melt pool near the laser head, with a sampling rate of 44.1 kHz that provided sufficient temporal resolution for detecting relevant acoustic phenomena associated with the DED-LB/M process [175]. However, no standardised guidelines have been published yet regarding the optimal positioning of microphones relative to the laser processing zone or the appropriate data acquisition rates.

Optical microphones are employed in applications requiring an extensive frequency bandwidth for signal acquisition in DED-LB/M monitoring, enabling the capture of acoustic responses over a spectrum ranging from 10 Hz to 1 MHz [179,180]. This wide frequency coverage allows for the capture of both low-frequency process variations and high-frequency emissions that are inherent to the DED-LB/M process. Research has shown that optical microphones can effectively acquire acoustic signals within the 10 Hz to 1 MHz range during DED-LB/M operations, confirming their ability to capture acoustic signatures associated with defects occurring during the fabrication of DED-LB/M structures [180].

It is important to note that while optical microphones offer superior frequency coverage, they are generally more expensive than traditional pre-polarised microphones. This cost factor may limit their widespread adoption in budget-constrained settings. In contrast, pre-polarised microphones, despite their more limited frequency range, have proven effective in capturing relevant acoustic phenomena at a more affordable price point. These conventional microphones typically operate in a narrower frequency band but still provide valuable insights into the DED-LB/M process.

##### Process Signatures

The process of extracting and analysing acoustic signatures is crucial for monitoring and understanding DED-LB/M processes, particularly in correlating acoustic features with process-induced defects. Initially, acoustic sensors capture raw signals that represent the voltage of acoustic far-field pressures over time, resulting in a time-domain signal [14]. This raw data, while informative, requires further processing to extract meaningful features that can provide insights into the DED-LB/M process dynamics and potential defect formation. Acoustic feature extraction is a key step in this process, transforming the raw signals into a set of characteristics that can be more easily interpreted and analysed, especially in relation to process-induced defects. These extracted features are typically classified into three main categories, as illustrated in Table 6. Time-domain features, derived directly from the signal’s amplitude variations over time, can indicate sudden changes or anomalies that might correspond to defect formation. Frequency-domain features reveal the signal’s spectral composition, typically obtained through Fourier transform techniques, which can highlight specific frequency signatures associated with different types of defects. Time-frequency domain features capture how the frequency content evolves throughout the process, providing a more detailed view of how different frequency components change over time during the DED-LB/M process [183]. Short-Time Fourier Transform (STFT) or continuous wavelet transforms (CWT) are often used to extract these features, allowing for the identification of transient events or localised defects that may occur at specific stages of the deposition process.

##### Analysis of Acoustic Features to Predict Defects

The processing and analysis of acoustic signals, along with the extraction of relevant acoustic features, play a crucial role in correlating these characteristics with various process-induced defects in DED-LB/M fabricated structures. Studies have shown that acoustic features contain valuable information for detecting defects in DED-LB/M fabricated structures, as summarised in Table 7, which outlines AE-based defect detection approaches in DED-LB/M without ML techniques, and Table 8, which focuses on AE-based detection approaches using ML techniques.

When a crack forms or propagates, it triggers a sudden release of stored elastic energy within the material. This abrupt energy release generates distinctive acoustic waves that can be captured and analysed. Consequently, distinct peak values in the acoustic energy serve as clear indicators of material fracture, with the energy release attributed to both initial crack formation and subsequent propagation. By analysing these characteristic patterns in the AE signals, it becomes possible to identify and potentially predict the occurrence of cracks and other structural defects in DED-LB/M fabricated structures.

The literature indicates that acoustic signals contain valuable information across various frequency ranges, particularly in the higher frequency band of 350 kHz to 1 MHz [169,179,180], as well as in the audible spectrum (20 Hz to 20 kHz) [175,177,182]. Several studies have attempted to identify the occurrence of cracks based on the acoustic energy within this specific higher frequency band [179,180]. It has been observed that AE signals often exhibit a sharp increase in acoustic energy corresponding to crack formation and propagation. While some methodologies have demonstrated effectiveness in detecting cracks based on acoustic energy analysis, differentiating cracks from disturbance events remains largely unexplored.

Several studies demonstrated that acoustic signals contain sufficient information in the audible frequency range (i.e., 20 Hz–20 kHz) to reliably characterise and distinguish between different process conditions [172] and process-induced cracks [175,177,182]. Hauser et al. [172] identified that the primary source of acoustic emissions in DED-LB/M was the interaction between the powder particles and the laser beam. Their research demonstrated the effectiveness of MFCCs analysis in detecting process instabilities during DED-LB/M fabrication. They identified two key frequency ranges: general process acoustic emissions below 600 Hz, which are present in both stable and unstable processes and likely correspond to background noise from powder delivery and equipment operation, and acoustic emissions between 2 kHz and 10 kHz, which are specifically associated with unstable processes. However, a critical limitation of their study is that it lacks correlation between the acoustic signatures and specific defect types, such as porosity or cracks.

Kim et al. [177] conducted a study to characterise the acoustic signatures associated with crack formation in DED-LB/M fabricated structures. By employing comprehensive signal analysis techniques across the time-domain, frequency-domain, and time-frequency domain, their results revealed that crack initiation produces a characteristic acoustic response, predominantly observed in the 12–16 kHz frequency band. The study further identified delayed cracking events, which occurred after the deposition process had finished. These delayed cracks were attributed to residual stresses within the fabricated structure and were detected through continuous acoustic monitoring post-fabrication. This finding highlights the importance of extended monitoring periods in DED-LB/M processes to capture all potential defect formations, including those that may develop over time due to internal stresses in the material. However, a notable limitation of this research is that it was conducted in a controlled lab environment, which restricts the applicability of its findings to real-world industrial settings. Additionally, the study lacks an exploration of methods to distinguish crack events from external disturbances through frequency spectrum analysis, which is crucial in noisy production environments.

Wu et al. [178] demonstrated the feasibility of using audible acoustic signals (10 Hz to 10 kHz) for identifying balling defects in DED-LB/M fabricated structures. By analysing the time and frequency domain characteristics of these signals, the researchers revealed strong correlations between these acoustic features and the occurrence of balling defects, showing that it’s possible to detect such defects during the DED-LB/M process. Importantly, their focus on lower frequencies may have excluded higher-frequency defect-related signals, such as those associated with cracking. Weber et al. [181,182] utilised audible acoustic signals to identify cracks and delamination defects in DED-LB/M fabricated structures. Through STFT and CWT-based analysis, they observed distinct frequency peaks between 11 and 18 kHz, with notable peaks at 11.5 kHz, 12.9 kHz, and 16 kHz corresponding to the occurrences of cracks and delamination in the fabricated structures. While these studies strengthen the viability of acoustic monitoring for defect detection, they share common limitations: both lack location-specific defect detection capabilities and have not been validated in industrial environments characterised by noisy acoustic conditions.

Recent developments in acoustic monitoring for DED-LB/M have seen the integration of ML approaches with advanced signal processing techniques to extract meaningful information from raw acoustic emissions. The extracted features are typically time-based, frequency-based, and time–frequency-based, and are subsequently used to train ML models for detecting and classifying defects including porosity and cracking in DED-LB/M components. For instance, Gaja et al. [169,171] employed several ML techniques, including K-Means clustering, logistic regression (LR), and artificial neural network (ANN) models, to analyse acoustic signals captured during the DED-LB/M fabrication process. Their developed technique successfully distinguished between acoustic signals generated by porosity and crack formations in DED-LB/M fabricated structures. Their findings revealed distinct characteristics for AE signals associated with different defect types. Acoustic signals generated by pore formation exhibited short decay times and relatively small amplitudes, while crack-related acoustic signals were characterised by short durations and high amplitudes. Taheri et al. [168] analysed acoustic emissions from the DED-LB/M process to assess their potential for identifying variations in manufacturing conditions. By employing a K-Means clustering approach, they were able to evaluate the degree of cohesion and separation within the signal clusters. The analysis revealed a strong association between the acoustic patterns and the integrity of the fabricated components. Their approach achieved predictive accuracy exceeding 87% across most operational scenarios. Li et al. [167] extracted time-frequency features from raw AE signals and applied various ML techniques, including K-means clustering, SVM, RF, and back propagation neural network, to classify operational conditions and detect undesirable deposition tracks. Their findings indicate a strong correlation between AE signals and operational conditions, with SVM achieving the highest classification accuracy of 94%.

In industrial manufacturing settings, the acoustic emissions produced during the DED-LB/M process comprise a blend of routine process signals, defect-induced events, and a range of extraneous sources. These interferences can originate from mechanical vibrations generated by the robotic laser head, the sound produced by shielding and carrier gas streams, operator activities near the build zone, and background noise from adjacent machinery. One of the main challenges in this field is to reliably detect authentic acoustic signatures linked to defect formation while distinguishing them from extraneous disturbances. Additionally, it requires establishing robust correlations between the derived acoustic signatures and the spatial occurrence of defects within DED-LB/M fabricated structures to demonstrate the successful application of the developed technique. To address this, Ansari et al. [14,36] developed an optimised AE signal processing methodology that analyses raw AE signals by calculating second-order derivatives and examining amplitude decay characteristics. This approach robustly filters out ambient noise and external disturbances, enabling the clear distinction of crack-related acoustic events. By leveraging these signal features, the method effectively correlates AE signals to the timing and precise location of cracks during the DED-LB/M process, providing a reliable and real-time detection capability even within complex industrial acoustic environments.

Researchers have initiated the development of sophisticated ML-based signal processing techniques to address the challenge of acoustic signal denoising in DED-LB/M processes, primarily within controlled laboratory environments. For instance, Chen et al. [173] proposed a deep learning–oriented methodology aimed at enhancing acoustic signal quality in DED-LB/M monitoring. The approach utilised a combination of audio equalisation, bandpass filtering, and Harmonic-Percussive Source Separation to suppress background disturbances and emphasise the acoustic features originating from the DED-LB/M process. This enabled the recovery of reliable signal characteristics, which were subsequently processed using MFCCs for CNN-based defect classification, achieving prediction accuracies of up to 93% for keyhole pore detection [175]. However, these advancements were validated primarily in controlled laboratory environments, raising concerns about their effectiveness in real-world industrial settings, which are often characterised by complex noise profiles that could significantly hinder the performance of such denoising techniques. 

To extend this work, Chen et al. [174] introduced a multi-sensor fusion digital twin (MFDT) that combined acoustic, visual, and thermal data for defect prediction. The system outperformed single-sensor methods, achieving 96% overall accuracy with a reduced false alarm rate of 4.4%. However, its predictions were restricted to classifying time intervals as defective, without identifying the type or quantity of individual defects, thereby limiting its resolution for detailed defect characterisation. 

The acoustic analysis based on time and frequency domain characteristics provides valuable information for detecting defects, but it falls short in pinpointing their exact locations in DED-LB/M fabricated structures. Therefore, alongside the acoustic defect detection system, there is a critical need to develop an acoustic source localisation system. This integrated approach would not only identify defects through acoustic signatures but also accurately determine their positions within the DED-LB/M fabricated structures. 

Weber et al. [22] proposed an approach to locate airborne acoustic sources using multi-sensor arrays, intending to apply this technique to locate defects in DED-LB/M fabricated structures. The researchers simulated acoustic events in a controlled laboratory environment using an omnidirectional speaker emitting a 12 kHz frequency band. The experimental setup consisted of a microphone array comprising six directional microphones integrated into the laboratory environment. The localisation technique utilised the TDOA method between acoustic signals to locate the speaker in the x- and y-directions. 

While initial results showed localisation errors up to 75 mm, the system demonstrated the capability to locate acoustic sources with errors as low as 3.9 mm in controlled laboratory conditions. However, despite this initial effort, the application of this acoustic source localisation technique using n-microphone arrays for pinpointing defects in actual DED-LB/M fabricated structures remains unexplored, both in laboratory and industrial settings.

##### Current Challenges, Existing Limitations, and Paths for Future Exploration

AE-based monitoring has demonstrated considerable potential for the detection of process-induced defects in DED-LB/M fabricated structures. The strong correlation between acoustic signatures and defect formation underscores the efficacy of AE techniques in identifying manufacturing anomalies during the DED-LB/M process, which is essential for ensuring quality assurance. The following section presents a comprehensive overview of the primary challenges encountered in this field. This analysis serves as a foundation for future research initiatives and technological advancements, providing valuable insights to guide upcoming investigations and foster innovation in acoustic-based monitoring for DED-LB/M processes.

1. The acoustic landscape of DED-LB/M operations is characterised by a complex interplay of multiple components, including normal process emissions, defect-related events, and various disturbances. This intricate acoustic environment presents significant challenges for accurate defect detection due to the presence of extraneous noise in the acoustic signals. Although researchers have employed frequency filtering and signal denoising techniques to address these challenges, there remains a pressing need for more advanced signal processing methods. These sophisticated approaches are necessary for effectively isolating defect-related acoustic signatures from the multitude of process-related sounds and external disturbances. The development of such advanced techniques is crucial for enhancing the reliability and precision of acoustic-based monitoring in DED-LB/M processes.
2. AE-based monitoring in DED-LB/M processes has demonstrated significant potential for defect detection and process characterisation, particularly in single-layer or single-track analyses. However, the layered nature of DED-LB/M fabrication introduces thermal complexities that significantly impact the acoustic signatures associated with defect formation and propagation. As successive layers are deposited, heat accumulation within the structure leads to dynamic changes in thermal gradients and stress distributions. Consequently, the acoustic landscape becomes increasingly complex when examining multi-layered structures, presenting new challenges for accurate defect identification and process monitoring. To address these challenges, a layer-by-layer analysis of acoustic signals becomes crucial. This approach allows for the examination of how acoustic signatures fluctuate throughout the build process, potentially revealing the formation of new defects or the propagation of existing ones within the fabricated structures.
3. AE-based analysis employing time and frequency domain characteristics has proven invaluable for defect identification in DED-LB/M processes. However, this approach exhibits inherent limitations in precisely localising defects within the fabricated structures. This limitation arises primarily from the focus of traditional acoustic analysis on identifying the occurrence of defects rather than their exact positioning. Consequently, the current methodologies, while effective in detecting defects, lack the capability to provide detailed spatial information about their locations within the fabricated structure. To address this shortcoming and enhance the overall effectiveness of acoustic monitoring in DED-LB/M processes, there is a critical need to develop an advanced acoustic source localisation system. Such a system would complement existing defect detection capabilities by providing location-specific information.

#### 3.2.5. Comparison of Different Sensing Methods

Table 9 summarises the various online process monitoring techniques, including vision sensing, thermal sensing, spectral sensing, and AE, utilised with ML algorithms for defect identification in DED-LB/M processes. Each of these methods offers distinct advantages and limitations that impact their effectiveness in real-time monitoring and defect detection.

**Vision and thermal sensing** techniques have demonstrated potential in detecting defects that occur in the surface and near-surface regions, particularly porosity and LoF. Vision sensing, which relies on the capture and analysis of melt pool images, provides direct observation of melt pool geometry. This approach has demonstrated efficacy in identifying anomalies and irregularities in the upper layers of fabricated structures. However, its effectiveness is limited to surface defects and can be compromised by variations in lighting conditions. Similarly, thermal sensing monitors temperature distribution and melt pool characteristics to detect thermal irregularities that may indicate defects. While this method has shown promise in identifying surface and near-surface defects based on thermal irregularities, it shares similar limitations with vision sensing in reliably detecting subsurface or internal defects, particularly cracks that may occur anywhere in the fabricated structures. Furthermore, both methods often require sophisticated ML algorithms for effective data analysis, adding a layer of complexity to their implementation in real-time industrial settings.

**Spectral Sensing** offers atomic-level information about the materials involved in the DED-LB/M process and has the capability to detect subsurface defects by analysing emitted light spectra. However, this method is sensitive to variations in deposition thickness and necessitates advanced data processing techniques to extract meaningful information from spectral data. The complexity involved in data interpretation can limit its practicality for real-time monitoring compared to other methods.

**AE monitoring** stands out for its ability to detect a wide range of defects, including both surface and subsurface defects. It shows promise for identifying interlayer cracks due to its broad defect detection capability. However, AE is highly sensitive to environmental noise, complicating the differentiation between actual defect signals and disturbances from external sources.

In conclusion, while each sensing technique presents distinct benefits, they also have inherent drawbacks that hinder their effectiveness in comprehensive defect detection. Despite advancements in these sensing methods, reliably detecting interlayer crack formation remains a significant challenge across all modalities.

### 3.3. Summary

Although notable advances have been achieved in developing online process monitoring techniques for DED-LB/M, critical research gaps persist in the reliable detection and characterisation of defects, particularly subsurface defects. Optical and infrared imaging approaches primarily concentrate on the melt pool and its immediate surroundings, limiting their effectiveness in detecting surface defects like porosity. However, these methods often overlook potential subsurface defects that may emerge following the addition of new layers or once the build is finalised. There is also, a lack of understanding about how the laser beam interacts with pre-existing defects and how this interaction influences defect propagation or reformation.

Vision- and thermal-based modalities provide only limited insight into the onset and progression of subsurface cracks and are insufficient for describing the mechanisms of crack reformation across multiple layers. This limitation highlights a critical need for layer-by-layer investigation to fully understand the defect initiation and propagation phenomena in subsequent layers. Such an approach would provide crucial insights into how defects evolve throughout the build process, how they interact with newly deposited material, and how they may influence the formation of new defects in subsequent layers. While spectroscopy-based methods promise to detect both surface and subsurface defects, including porosity, they still face challenges in providing comprehensive defect characterisation across multiple layers.

Multi-camera systems, while providing comprehensive monitoring, face challenges of high cost, hardware complexity, and calibration demands. These limitations restrict their practical deployment in industry. Future research should focus on simplifying hardware by developing multifunctional smart cameras that combine sensing modalities, reducing the number of cameras required. Strategic camera placement optimized through advanced calibration and photogrammetry techniques can maintain accuracy with fewer cameras. On the software side, leveraging AI-driven data fusion and machine vision algorithms can enable comprehensive defect characterization from limited camera views, reducing computational burdens while maintaining data quality.

The AE technique shows promise in detecting both surface and subsurface defects, particularly cracks, in DED-LB/M processes. However, the application of this technique faces significant challenges, especially in industrial environments where the acoustic landscape is complex. Most existing methods have focused on identifying defects in DED-LB/M fabricated structures produced under controlled laboratory conditions. Factors such as machine vibrations, ambient noise, and electromagnetic interference pose substantial challenges to AE-based defect detection in an industrial environment. More advanced signal processing techniques remain critical to effectively isolate defect-related acoustic signatures from process-related sounds and external disturbances.

Additionally, most AE methods and existing approaches often predict entire regions as flawed whenever a defect is detected, lacking accuracy in distinguishing between various defect categories (e.g., cracks or porosity) or providing clear information about the number and spatial distribution of defects within the fabricated structures. This limitation significantly hinders the ability to implement targeted interventions or optimise the process in real-time.

Furthermore, limited research can provide information about the mechanisms of defect reformation across successive layers. This limitation highlights a critical need for layer-by-layer investigation to fully understand the defect initiation and propagation phenomena in subsequent layers. Traditional methods of defect localisation often rely on post-process inspection techniques, for instance X-ray computed tomography or destructive testing. However, these methods are time-consuming and costly and do not allow real-time process intervention. Pinpointing the exact location of defects within the fabricated structure is crucial for understanding defect formation mechanisms and implementing targeted corrective actions. While AE techniques have shown potential for defect detection, their application to acoustic source localisation in DED-LB/M processes remains largely unexplored. To date, no studies have attempted to locate defects in DED-LB/M fabricated structures in either laboratory or industrial environments using real-time acoustic monitoring during the fabrication process, representing a significant gap in the current research. This lack of real-time defect localisation capability is a critical limitation in DED-LB/M process monitoring. Developing methods for accurate acoustic source localisation could deliver real-time information regarding the integrity of the fabrication process, allowing for on-the-fly modifications to processing variables to mitigate defect development or propagation.

## Figures and Tables

**Table 1:** Advantages and disadvantages of camera setups and filtering strategies in DED-LB/M monitoring.

Setup/Strategy	Advantages	Disadvantages	References
Co-axial Camera	Directional independence; undistorted, top-down views; accurate horizontal melt pool measurement.	Complex optics; sensitive to plume interference; limited vertical dimension capture.	[114,120]
Off-axis Camera	Simpler setup; useful side view; layer height and melt pool profile.	Single field of view; geometric distortion needing calibration; directional limits.	[58,113]
Dual/Multi-camera Setup	Complementary views; more complete melt pool info; enhanced defect detection.	Higher cost/complexity; data synchronisation and integration challenges.	[116,121]
Filtering Strategies	Improved image clarity; sensor protection; enhanced temperature measurement.	Possible signal loss; limited spectral range.	[117,118]

**Table 3:** Summary of thermal sensing-based defect detection approaches in the DED-LB/M process.

Process	Devices Used	Extracted Features (Input)	ML Algorithms	Defect Detection	Performance Indicators	Ref.
DED-LB/M	IR thermal camera (Off-axis) Pyrometer (Co-axial)	Morphological features of melt pools (e.g., area, length, width, etc.). Melt pool temperature distribution.	DT, KNN, SVM, LDA, QDA SOMs	Porosity	Recall—98.44% Accuracy—96%	Khanzadeh et al. [147]
	IR thermal camera (Off-axis) Pyrometer (Co-axial)	Melt pool temperature distribution (mean and standard deviation)	SVM	Porosity	Accuracy—90%	Gaikwad et al. [152]
	IR thermal camera (Off-axis) Pyrometer (Co-axial)	Morphological features of melt pools (e.g., area, length, width, etc.). Melt pool temperature distribution.	CNN, LRCN	Porosity	Accuracy—96%	Tian et al. [151] Guo et al. [155]
	Pyrometer (Co-axial)	Morphology dynamics of melt pools and HAZs	SVM	Porosity	Accuracy—96%	Bappy et al. [145]
	IR thermal camera (Off-axis) Pyrometer (Co-axial)	Morphological features of melt pools (e.g., area, length, width, etc.). Melt pool temperature distribution.	K-means clustering SOM	Porosity	Accuracy—97%	Ouidadi et al. [145]
	IR thermal cameras (Off-axis and Coaxial)	Morphological features of melt pools (e.g., area, length, width, etc.).	Statistical Analyses	Porosity	Undetermined	Herzog et al. [121]
	IR thermal cameras (Off-axis)	Melt pool temperature distribution.	Statistical analyses, Analysis of variance (ANOVA)	Process-induced defects	Undetermined	D’Accardi et al. [150]
	IR thermal cameras (Off-axis)	Melt pool temperature distribution.	Statistical Analyses	Crack	Undetermined	Mazzarisi et al. [146]

**Table 4:** Summary of spectra sensing-based defect detection approaches in the DED-LB/M process.

Process	Devices Used	Extracted Features (Input)	ML Algorithms	Defect Detection	Performance indicators	Ref.
DED-LB/M	Spectrometer	Spectral features such as emission energy of Al & Mg lines, line-to-continuum ratio etc.	LSTM-Autoencoder, K-means clustering	Porosity	Undetermined	Mazumder et al. [160]
	Spectrometer	Spectral features such as line-to-continuum ratio, average raw spectra intensity, background-subtracted emission line intensities, etc.	RF	Porosity	Accuracy—83%	
	ICP® Microphone Optical Emission Spectroscopy (OES) Sensors	Time domain & frequency domain characteristics, intensity of all spectra	LDA, LogReg, LSVM, LinearSVM, SVM, kNN, MLP, RF	Conduction mode, Lack-of- fusion	Accuracy (AE)—73.3% Accuracy (OES)—90%	Wasmer et al. [158]
	Spectrometer	Spectral intensity of different wavelength	Statistical process control	Process defects	Undetermined	Chen et al. [164]
	Spectrometer	Plasma RMS signal	Multiplicative scatter correction	Variations in the gas or powder flow	Undetermined	Valdiande et al. [161]

**Table 5:** Illustration of acoustic sensors used for in situ monitoring in DED-LB/M process, highlighting their device types and operational frequency ranges.

In situ Monitoring Method	Devices Used		Frequency Range
Acoustic Emission	Structure-borne acoustic sensors	Piezoelectric sensor (i.e., AE Sensors) [167,168,169,170,171]	50–130 kHz [167] 100–1000 kHz [169,171] 1.0 kHz–1.0 MHz [170]
	Air-borne acoustic sensors	ICP^®^ Microphone [172,173,174,175,176,177]	10 Hz–10 kHz [178] 50 Hz–20 kHz [172,173,174,175,176] 6.3–20 kHz [177]
		Optical Microphone [179,180]	10 Hz–1 MHz [179,180]
		Directional Microphone [181,182]	40 Hz to 20 kHz [181,182]

**Table 6:** Summary of extracted acoustic features for defect identification in DED-LB/M process.

Feature Type	Feature Name	Description	Reference
Time- domain	Mean	The average amplitude of the acoustic signal over time.	[167]
	Peak Amplitude	The maximum amplitude value (*x_i_*) within the acoustic signal window, indicating the loudest point in the signal during that time frame.	[167]
	Absolute mean	The average of the absolute amplitude values, indicating the signal’s magnitude.	[167]
	RMS (Root Mean Square)	The square root of the mean of squared amplitudes, representing signal strength.	[167]
	Absolute Std	The standard deviation of absolute values, showing variability in amplitude.	[167]
	Std of Envelope Lines	Standard deviation of the upper and lower amplitude envelopes of the signal.	[167]
	Kurtosis	A measure of the sharpness of the signal distribution, identifying extreme peaks.	[167,169,171]
	Skewness	Describes the asymmetry of the signal amplitude distribution around its mean.	[167]
	Acoustic Energy	The average power of the acoustic signal over time.	[167,169,171]
	Number of Counts	Number of times the signal crosses a preset threshold.	[169,171]
	Duration	Total time length of the acoustic event.	[169,171]
	Rise Time	Time from the start of the event to the peak amplitude.	[169,171]
Frequency- domain	Peak Amplitude Frequency	Frequency at which the maximum amplitude occurs in the spectrum.	[169,171]
	Spectral Centroid	The “centre of mass” of the signal’s spectrum, indicating dominant frequency regions.	[167]
	Spectral Skewness	A measure of asymmetry in the signal’s power spectrum, showing spectral bias.	[167]
	Spectral Kurtosis	A measure of the peakedness in the frequency domain, highlighting sharp spectral features.	[167]
Time-frequency domain	Short-time Fourier transform (STFT)	Represents how frequency content changes over time.	[174,175,181,182]
	Continuous Wavelet transforms (CWT)	Provides multi-resolution analysis of non-stationary signals.	[181,182]
	Mel-frequency Cepstrum Coefficients (MFCCs)	Represent the short-term power spectrum through a linear cosine transformation of a logarithmic power spectrum mapped onto a nonlinear mel frequency scale.	[174,175]

**Table 7:** Summary of AE-based defect detection approaches in the DED-LB/M process (without applying ML technique).

Process	Devices Used	Frequency Range	Extracted Features	Analysis Methods	Defect Detection	Ref
DED-LB/M	Optical Microphone	10 Hz–1 MHz	Acoustic energy	Peak values in acoustic energy	Crack	García de la Yedra et al. [180]
	Optical Microphone	10 Hz–1 MHz	STFT based spectrograms, acoustic energy	Crack detection based on the frequency range, peak values in acoustic energy	Crack, identified crack frequency range, (350 kHz–1 MHz)	Camilo et al. [179]
	ICP® Microphone	50 Hz–20 kHz	Mel frequency spectrum	Process condition detection based on the frequency range	Unstable process condition monitoring, frequency range (2 kHz and 10 kHz)	Hauser et al. [172]
	ICP® Microphone	6–20 kHz	Time-domain, frequency-domain, and time-frequency domain characteristics	Crack detection based on the frequency range	Crack, identified crack frequency range, (12 kHz–16 kHz)	Kim et al. [177]
	ICP® Microphone	10 Hz –10 kHz	Time and frequency domain characteristics	Balling detection based on the frequency range	Balling	Wu et al. [178]
	Directional Microphone	40 Hz –20 kHz	STFT and CWT based spectrograms	Crack and delamination detection based on the frequency range	Crack and delamination. Identified defects frequency range, (11 kHz–18 kHz)	Weber et al. [182]

**Table 8:** Summary of AE-based defect detection approaches in the DED-LB/M process (using ML technique).

Process	Devices Used	Frequency Range	Extracted Features (Input)	ML Algorithms	Defect Detection	Performance Indicators	Reference
DED-LB/M	AE Sensors	100–1000 kHz	Time domain & frequency domain characteristics	K-means clustering, LR, and ANN	Crack, Porosity	Accuracy—85.7%	Gaja et al. [169,171]
	Piezoelectric sensor	-	Time domain & frequency domain characteristics	K-means clustering	Classification of different process conditions	Accuracy—87%	Taheri et al. [168]
	Piezoelectric sensor	50–1300 kHz	Time domain & frequency domain characteristics	K-means clustering, SVM, RF, and back propagation neural network (BPNN)	Identification of different operation conditions Powder feeding situation, laser melting situation, abnormal and normal deposition	Accuracy from 88 to 94%	Li et al. [170]
	ICP® Microphone	50 Hz–20 kHz	MFCCs, STFT based spectrograms	CNN architecture	Keyhole pore, crack	Accuracy: 89%, Keyhole pore accuracy: 93%	Chen et al. [174,175]

**Table 9:** Comparison of various real-time monitoring methods used for identifying defects during the DED-LB/M process.

Monitoring Method	Monitored object	Main ML Algorithms	Defects			Advantages	Disadvantages
			Crack	Porosity	Lack-of-Fusion		
Vision Sensing	Melt pool geometrical characteristics	ANN, SVM, RF, D-CNN	×	√	√	Direct observation of melt pool geometry.	Limited to identify surface defects.
Thermal Sensing	Melt pool geometrical characteristics and temperature distribution	K-means clustering, ANN, SVM, RF, CNN, LRCN	√	√	×	Provides real-time information on melt pool thermal characteristics.	Requires accurate emissivity calibration. Limited to identifying surface and near-surface defects.
Spectral Sensing	Spectral features	K-means clustering, RF, KNN, SVM	×	√	√	Provides atomic-level information about the process. Can detect subsurface defects.	Sensitive to deposition thickness variations. Requires advanced data processing to extract meaningful information.
Acoustic Emission	Acoustic signal	K-means clustering, ANN, SVM, RF, CNN	√	√	×	Can detect surface and subsurface defects.	Sensitive to external disturbances and environmental noise

## Data Availability

No new data were created or analysed in this study. Data sharing is not applicable to this article.
